# Prehospital Risk Stratification Using Unsupervised Machine Learning in STEMI

**DOI:** 10.1111/eci.70214

**Published:** 2026-04-24

**Authors:** Ana Ramos‐Rodríguez, Raúl López‐Izquierdo, Carlos del Pozo Vegas, María Plaza‐Martín, Cristina Tapia‐Ballesteros, Juan F. Delgado Benito, Ancor Sanz‐García, Francisco Martín‐Rodríguez

**Affiliations:** ^1^ Emergency Department Hospital Universitario Rio Hortega Valladolid Spain; ^2^ Faculty of Medicine Universidad de Valladolid Valladolid Spain; ^3^ CIBER of Respiratory Diseases (CIBERES) Institute of Health Carlos III Madrid Spain; ^4^ Valladolid Health Research Institute (IBioVALL) Valladolid Spain; ^5^ Emergency Department Hospital Clínico Universitario Valladolid Spain; ^6^ Cardiology Department Hospital Clínico Universitario de Valladolid Valladolid Spain; ^7^ CIBER of Cardiovascular Diseases (CIBERCV) Institute of Health Carlos III Madrid Spain; ^8^ Cardiology Department Hospital Universitario Rio Hortega Valladolid Spain; ^9^ Emergency Medical Services (SACYL) Valladolid Spain; ^10^ Faculty of Health Sciences Universidad de Castilla La Mancha Talavera de la Reina Spain; ^11^ Technological Innovation Applied to Health Research Group (ITAS Group), Faculty of Health Sciences University of de Castilla‐La Mancha Talavera de la Reina Spain; ^12^ Health Care Evaluation Research Group (ECUSAL) Instituto de Investigación Sanitaria de Castilla‐La Mancha (IDISCAM) Toledo Spain

**Keywords:** clinical decision making, machine learning, phenotype, prehospital care, STEMI

## Abstract

**Background:**

ST‐elevation myocardial infarction (STEMI) exhibits substantial clinical heterogeneity complicating prehospital risk stratification. Traditional risk assessment tools often fail to capture the complexity of this condition. Machine learning offers opportunities to identify complex clinical patterns not readily apparent during prehospital care.

**Aim:**

To identify distinct phenotypes in STEMI patients using unsupervised machine learning algorithms based on prehospital parameters, and to determine their association with short‐term mortality and cardiovascular outcomes.

**Methods:**

Prospective multicenter observational cohort study including adult patients with prehospital STEMI code activation transported by emergency medical services from January 2022 to August 2025. Only EMS‐transported patients were included; those who self‐presented to the emergency department were excluded. Prehospital variables, including demographic, clinical, and procedural data, were used for clustering. Factor Analysis of Mixed Data and a two‐step clustering: hierarchical clustering (exploring structure and number of clusters) and k‐means (clustering assigning patients to phenotypes). A Random Forest classifier with SHapley Additive exPlanations values was used to identify variables contributing to cluster assignment. The primary outcome was 30‐day all‐cause mortality, assessed through follow‐up records.

**Results:**

Among 744 patients (median age, 65 years; 76.3% male) unsupervised clustering identified three distinct phenotypes: Phenotype‐1 (70.3%) characterized by hemodynamic stability, vessel locations, Killip class I presentation (70.6%), and favourable laboratory parameters; Phenotype‐2 (24.3%) presented higher comorbidity burden and metabolic derangements; and Phenotype‐3 (5.4%) exhibiting profound hemodynamic instability, severe respiratory failure, out‐of‐hospital cardiac arrest with return of spontaneous circulation (87.5%), Killip class IV presentation (67.5%), and marked metabolic derangements. The 30‐day mortality rates were: 3.4% in Phenotype‐1, 22.1% in Phenotype‐2, and 75.0% in Phenotype‐3.

**Conclusions:**

Three clinically distinct STEMI phenotypes were identified with markedly different mortality risks and treatment requirements during prehospital care. Phenotypes derived from readily available prehospital parameters may facilitate early risk stratification, optimize triage decisions, and guide individualized therapeutic strategies.

## Graphical abstract

Machine learning identified three phenotypes with 30‐day mortality of 3.4%, 22.1%, and 75%. Phenotype‐2 reveals an "occult high‐risk" group: hemodynamically stable yet six‐fold higher mortality.

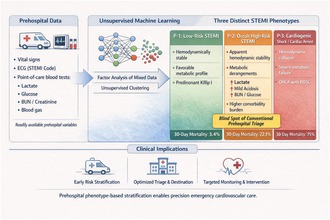

## 1 Introduction

ST‐segment elevation myocardial infarction (STEMI) remains a leading time‐sensitive cardiovascular emergency worldwide, accounting for 25%–40% of all acute coronary syndromes. Despite considerable advances in therapeutic strategies, STEMI continues to impose substantial morbidity and mortality burdens on healthcare systems globally [1]. The paradigm that “time is muscle” has fundamentally transformed the management of this condition, establishing prompt reperfusion therapy as the cornerstone of treatment. In this context, every minute of delay translates into irreversible myocardial loss, directly impacting patient survival and long‐term clinical outcomes [2].

The clinical heterogeneity of STEMI presents a substantial challenge for emergency medical services (EMS). Patient presentations span a broad spectrum, from classic anginal chest pain to atypical or silent manifestations, particularly among vulnerable populations including individuals with diabetes mellitus, elderly patients, and women [3]. This phenotypic variability not only complicates timely diagnosis but also profoundly influences the risk of acute complications, therapeutic response, and both short‐ and long‐term cardiovascular outcomes. Recognition of these diverse clinical presentations is essential for optimizing triage protocols and reducing time‐to‐treatment intervals [4].

Indeed, the inherent heterogeneity of STEMI necessitates risk stratification approaches that are more refined than traditional criteria based solely on electrocardiographic location, age, or comorbidities [5]. Artificial intelligence and data analytics have created novel opportunities to identify complex clinical patterns that may not be readily apparent to EMS providers at the scene or en route [6]. Machine learning (ML) via unsupervised clustering represents an innovative approach to identify patient subgroups with similar clinical characteristics without requiring prior knowledge of classification categories [7].

Consequently, the objective of the present study was to identify distinct phenotypes in patients with STEMI using unsupervised clustering techniques, incorporating clinical, demographic, electrocardiographic, and comorbidity variables collected in prehospital care. Second, to evaluate the association between the identified phenotypes and the risk of acute complications, therapeutic response, and short‐ and long‐term cardiovascular outcomes, with the aim of optimizing risk stratification and treatment in the prehospital care.

## 2 Methods

### 2.1 Study Design and Settings

A prospective, multicenter, EMS‐delivered, ongoing study was conducted in patients with STEMI code activation by the EMS system and transported with high priority by ambulance to the emergency department (ED). Patients were recruited consecutively and prospectively (24/7/365) from the “Advanced Precision Scoring‐System for Prehospital Critical Care Based on Artificial Intelligence” (APPS study) conducted across three Spanish EMS systems: SACYL (Castile and León Health Service), Emergentziak‐Osakidetza (Basque Health Service), and SESCAM (Castile‐La Mancha Health Service), serving a population of 3,931,331 inhabitants, from January 1, 2022, through August 30, 2025. Although the APPS study remains ongoing, the dataset used for the present analysis was formally closed on August 30, 2025 (data freeze date) and no further patient recruitment was performed after this date for the current report.

The study included 36 advanced life support (ALS) units, 7 helicopter emergency medical services (HEMS), 3 emergency dispatch centers, and 29 hospitals (11 tertiary university hospitals and 18 small general district hospitals with metropolitan and rural distribution). ALS units are staffed with a registered nurse (RN), a physician, and two emergency medical technicians (EMTs). HEMS units are crewed by an RN, a physician, a pilot, and a flight mechanic. The healthcare systems participating in the study (including operational coordination centers, EMS systems, and EDs) operate under comparable protocols and similar workflows in accordance with international standards for advanced life support.

The 11 tertiary university hospitals have 24‐h cardiac catheterization laboratory capabilities. For the small general district hospitals, when percutaneous coronary intervention (PCI) is required, the EMS system performs emergent transport (preferably by air) to the nearest appropriate facility within 90 min.

The study was approved by the institutional review board of the Public Health Service (reference: 23‐PI027, PI217‐20, and EAG‐PRE‐2024‐01). This study follows the Strengthening the Reporting of Observational Studies in Epidemiology (STROBE) guidelines (Supporting Information p3).

### 2.2 Population

Adult patients (≥ 18 years) with a prehospital STEMI code and subsequently transferred to the ED were consecutively included in the study. Activation criteria for the prehospital STEMI code included: chest pain or symptoms suggestive of ischemia lasting ≥ 20 min in duration, ST‐segment elevation, measured at the J point, in two contiguous leads ≥ 0.1 mV (except in V2 and V3 where the required elevation varies according to the patient's age and sex), new or presumably new complete left bundle branch block or right bundle branch block, and paced ventricular rhythm [8]. The final sample included cases of prehospital STEMI code activation that were ultimately confirmed as STEMI by the cardiology specialist at the hospital level.

Minors, pregnant women (evident or suspected), witnessed out‐of‐hospital cardiorespiratory arrest (OHCA) not recovered on‐scene, patients in end‐stage condition documented by specialist report, unavailability of prehospital blood tests (e.g., failure of point‐of‐care testing, inaccessible venous line blood collection), or lack of informed consent were excluded.

### 2.3 Outcomes

The primary outcome was 30‐day in‐hospital mortality, consistent with comparable studies, validated outcome, and widely used to evaluate the impact of interventions in patients with STEMI [9].

Secondary prehospital outcomes included: mechanical ventilation, electrical therapy (defibrillation, cardioversion, and external pacemaker), drug administration, and witnessed OHCA with return of spontaneous circulation (ROSC). Hospital outcomes comprised: fibrinolysis, PCI, emergency surgery, mechanical ventilation, vasoactive agents, primary STEMI location, multivessel involvement, Killip classification, length of hospital stay, and 2‐day mortality.

### 2.4 Data Collection

In‐person training was provided for both EMS providers and ED providers. The mandatory training included setup and operation of the point‐of‐care testing (POCT), cleaning, documentation, and troubleshooting, as well as proper completion of the digital data collection log.

Data collection employed a dual‐entry protocol within a custom‐designed database, capturing both prehospital metrics and longitudinal hospital outcomes. To establish reliable patient matching between EMS documentation and hospital electronic health records (EHRs), a minimum of five concordant identifiers were required from the following set: ED arrival time, biological sex, age, EMS incident identifier, given name, family name, and health insurance number. Records lacking sufficient matching criteria were systematically excluded from analysis. Database security incorporated password protection coupled with two‐factor authentication protocols. Following initial tabulation and quality control procedures, the data manager (ASG) executed comprehensive de‐identification of all protected health information.

Data collection was a four‐step process. First, epidemiological variables (age, sex at birth, urban or rural area, night shift, and weekend) and basal vital signs (oxygen saturation, blood pressure, heart rate, and temperature) via a LifePAK 15 monitor‐defibrillator (Physio‐Control Inc., Redmond, USA), as well as respiratory rate, fraction of inspired oxygen (F_I_O_2_), and Glasgow Coma Scale (GCS) were collected from the RN. The oxygen saturation/fraction of inspired oxygen ratio (SaFi), mean blood pressure (MBP), and thrombolysis in myocardial infarction (TIMI) risk index (TRI) were subsequently calculated (Table S1). Second, the RN proceeded to establish peripheral vascular access and obtained a venous blood sample in a 2 mL syringe that was subsequently analysed via the Epoc Blood Analysis System (Siemens Healthcare GmbH, Erlangen, Germany) to obtain within 3 min: complete blood gas, electrolytes, haemoglobin, haematocrit, glucose, lactate, creatinine, urea, and blood urea nitrogen (BUN). In a third step, and still in the prehospital care setting, the physician collected the baseline electrocardiographic rhythm, STEMI activation criteria, comorbidities, and risk factors, and upon arrival at the critical care bay, the first medical contact‐to‐balloon time (arrival cardiac catheterization laboratory). Finally, an ED provider completed the 30‐day follow‐up, collected the hospital outcome data, and recorded 2‐ and 30‐day mortality rates.

### 2.5 Data Analysis

**FIGURE 1:**
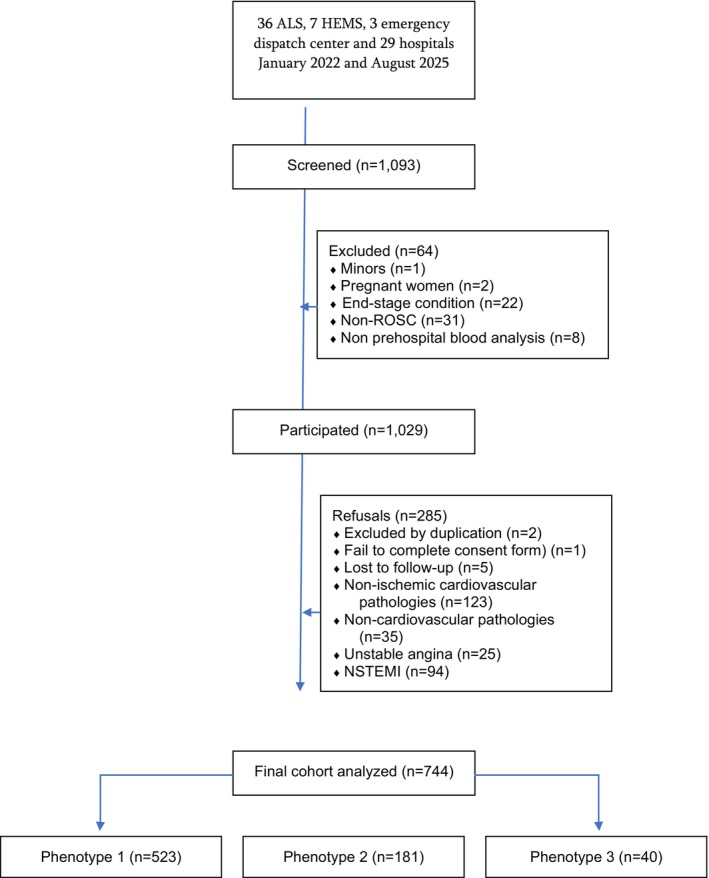
Study flowchart. ALS, advanced life support unit; HEMS, helicopter emergency medical service; NSTEMI, non‐ST‐elevation acute coronary syndrome; ROSC, recovery of spontaneous circulation.

Unsupervised clustering was performed to identify distinct patient phenotypes based on all baseline variables, which included a mixture of numerical and categorical data. Note that the only missing data concerned follow‐up information in five patients (Figure 1). Factor Analysis of Mixed Data (FAMD) was applied to reduce dimensionality and summarize the original variables in continuous components [10] (the first 10 FAMD dimensions were retained for clustering, explaining approximately 40% of total variance. This approach was chosen to preserve secondary sources of variability that may contribute to clinically meaningful phenotypic separation. The robustness of the clustering solution was subsequently confirmed through multiple internal validation procedures [hierarchical structure, silhouette analysis, and consensus clustering]). Clustering was performed in a two‐step approach. First, hierarchical clustering using Ward's method was applied to the Euclidean distance matrix of the retained FAMD components to explore the structure of the data and inform the optimal number of clusters. The dendrogram suggested a three‐cluster solution, which was further supported by silhouette analysis. Subsequently, k‐means clustering (k = 3, nstart = 25) was performed on the FAMD coordinates to obtain the final phenotype assignments. Although the average silhouette width peaked at K = 2, the two‐cluster solution merged patients with distinct clinical profiles (hemodynamically stable with high comorbidity burden versus overtly unstable with cardiac arrest) into a single undifferentiated high‐risk group, obscuring clinically meaningful heterogeneity. The three‐cluster solution was preferred because it separated an intermediate “occult high‐risk” phenotype (P‐2)—characterized by preserved hemodynamics but biochemical hypoperfusion and high comorbidity burden—from the overtly critical phenotype (P‐3), a distinction with direct therapeutic implications. This decision was supported by the dendrogram structure and validated by the consensus clustering results (ARI = 0.927). To identify variables contributing most strongly to cluster assignment, a Random Forest classifier (implemented using the ranger package; 100 trees; probability estimation enabled, mtry: square root of the number of predictors (default setting), Minimum node size: default (1 for classification), Splitting rule: Gini impurity, and Sampling: bootstrap sampling without replacement) was fitted with cluster membership as the dependent variable and the original feature set as predictors, and SHAP values were computed to quantify feature importance. Given that the primary purpose of the Random Forest model in this study was not predictive optimization but post hoc interpretability of cluster assignments (via SHAP values), we opted for standard/default hyperparameters to avoid overfitting and to maintain model stability and reproducibility. Hyperparameter tuning can improve predictive performance; however, in this context, maximizing classification accuracy was not the primary objective. Instead, model interpretability and robustness were prioritized. The data analysis flowchart can be found in Figure S1. Descriptive statistics and between‐cluster comparisons were then performed using standard methods, in which continuous variables were expressed as median (interquartile range, IQR) and categorical variables were expressed as absolute value and percentage, *N* (%), were used to present the resulting clusters. The comparisons between clusters were performed via Kruskal–Wallis, Dunn pairwise test, and Chi‐square. A *p*‐value < 0.05 was considered statistically significant after adjusting for multiple comparison. Data collection, missing value handling and sample size calculations can be found in Supporting Information p5. All analyses were conducted in R version 4.2.2 (http://www.R‐project.org; the R Foundation for Statistical Computing, Vienna, Austria), using FactoMineR, factoextra, ranger, fastshap, and base R functions.

## 3 Results

The EMS performed 1029 activations of the STEMI Code; of these, 123 were other acute non‐ischemic cardiovascular pathologies, 35 cases presented other acute non‐cardiovascular pathologies, 25 unstable angina, 94 non‐ST‐elevation acute coronary syndrome (NSTEMI), and finally 744 cases of STEMI that comprised the final analysis (see Figure 1). The median age was 66 years (IQR: 57–76; range 31–103), and 23.8% (177 cases) were female. The 30‐day cumulative in‐hospital mortality (all‐cause) was 11.8% (88 cases), and 2‐day mortality was 7.3% (54 cases).

Phenotyping based on unsupervised ML criteria revealed three well‐defined clinical clusters: P‐1 with 523 patients, P‐2 with 181, and P‐3 with 40 patients. The percentage of explained variances by dimensions of the FAMD analysis is shown in Figure S2, as well as the optimal number of clusters (Figure S3) and the dendrogram resulting from the hierarchical clustering (Figure S4).

**FIGURE 2:**
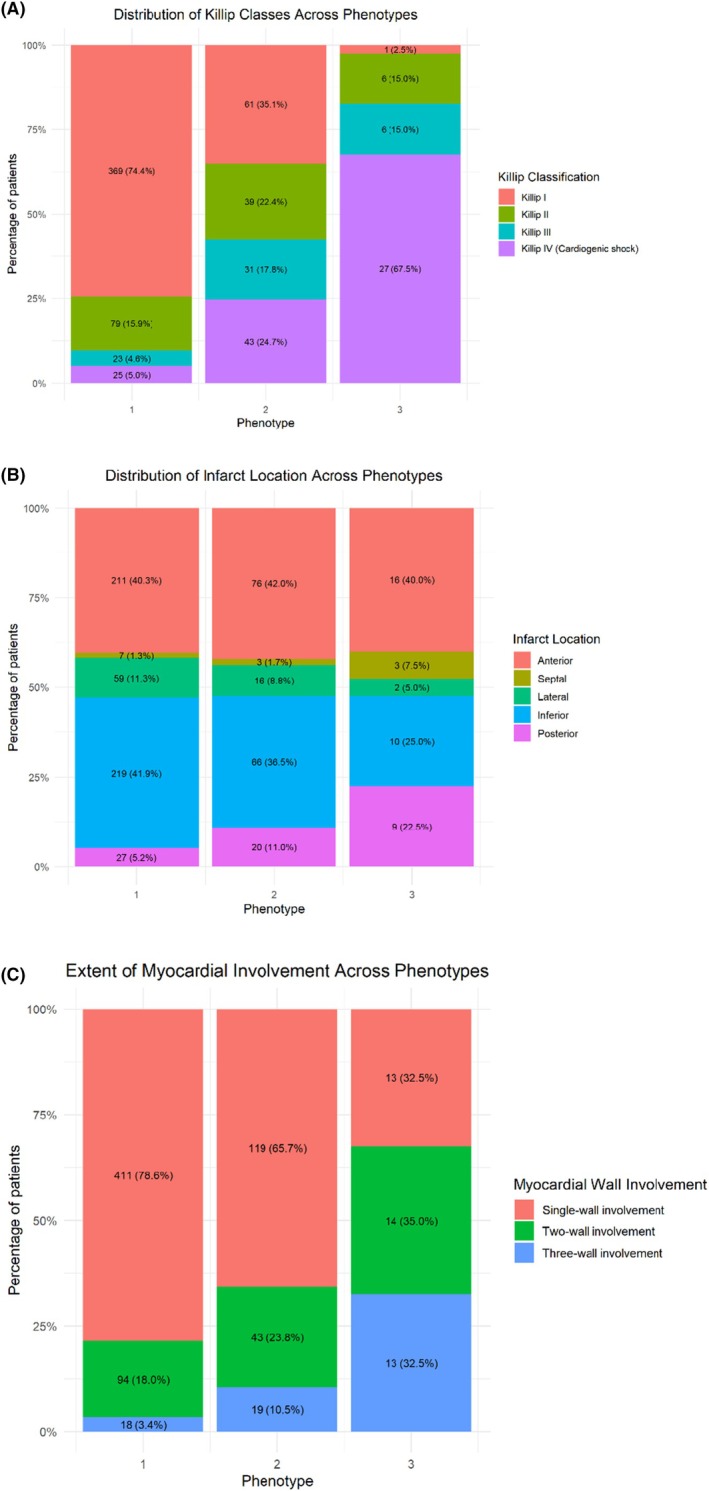
Distribution of patients according to relevant cardiological characteristics: (A) Killip classification, (B) Primary STEMI location, and (C) Multivessel disease.

The epidemiological criteria, vital signs, biomarkers, comorbidities, and risk factors used to derive the phenotypes can be observed in Table 1. Phenotype P‐1 comprised 70.3% (523 cases), with a 30‐day mortality rate of 3.4% (18 cases) and a 2‐day mortality rate of 1.3% (7 cases). A total of 41.9% (219 cases) had an inferior vessel location, and 70.2% (369 cases) had Killip classification class I (Figure 2A–C). As expected, this phenotype presented subtle alterations in vital signs and laboratory data, with the lowest rate of interventions both in the prehospital and in‐hospital settings, with dyslipidemia, hypertension, and smoking use being the most notable comorbidities and a median first medical contact (FMC)‐to‐balloon time of 55 min (IQR: 41–73).

**TABLE 1:** Baseline patient characteristics of primary coded patients with novel STEMI according to machine‐learning cluster analysis.

	Unsupervised clustering phenotypes			*p* ^b^
	P‐1	P‐2	P‐3	
No. (%) with data^a^	523 (70.3)	181 (24.3)	40 (5.4)	N.A.
Age	64 (55–71)	75 (65–85)	68 (58–79)	< 0.001
Sex, female	112 (21.4)	55 (30.4)	10 (25)	0.051
Basal vital signs				
Respiratory rate, breaths/min	17 (14–19)	17 (14–20)	8 (6–21)	< 0.001
Oxygen saturation, %	97 (95–99)	95 (91–98)	75 (57–87)	< 0.001
Fraction of inspired oxygen, %	0.21 (0.21–0.21)	0.21 (0.21–0.21)	0.21 (0.21–0.5)	< 0.001
SaFi	462 (452–471)	443 (398–466)	267 (130–379)	< 0.001
Systolic blood pressure, mmHg	140 (120–158)	121 (103–141)	82 (62–97)	< 0.001
Diastolic blood pressure, mmHg	85 (72–96)	70 (59–80)	43 (31–57)	< 0.001
Mean blood pressure, mmHg	103 (90–115)	87 (73–100)	56 (41–70)	< 0.001
Heart rate, beats/min	74 (62–87)	79 (60–100)	108 (60–173)	< 0.001
Temperature, °C	36 (36–36.3)	36 (35.8–36.1)	36 (35.1–36.5)	0.008
Glasgow coma scale, points	15 (15–15)	15 (15–15)	3 (3–5)	< 0.001
TIMI risk index	20.9 (14.8–28.6)	36.2 (26.1–50.6)	52.3 (36.7–85.6)	< 0.001
Prehospital blood test				
pH	7.39 (7.36–7.43)	7.34 (7.28–7.39)	6.99 (6.88–7.11)	< 0.001
pCO2, mmHg	40 (34–45)	43 (34–50)	56 (49–74)	< 0.001
pO2, mmHg	32 (23–44)	23 (21–32)	16 (11–22)	< 0.001
Bicarbonate, mEq	23.6 (21.9–26.4)	21.6 (20.1–24.6)	15.4 (13.2–17.4)	< 0.001
Base excess, mmol/L	0.7 (−1.2; 1.7)	−1.6 (−3.7; 1)	−12.7 (−15.6; −6.9)	< 0.001
cSO2, %	49 (38–74)	41 (31–54)	22 (15–32)	< 0.001
Sodium, mmol/L	140 (137–141)	140 (136–141)	140 (137–141)	0.287
Potassium, mmol/L	4 (3.7–4.3)	4.1 (3.8–4.7)	5.6 (4.6–6.6)	< 0.001
Calcium, mmol/L	1.14 (1.08–1.22)	1.14 (1.08–1.22)	1.14 (1.01–1.23)	0.207
Chlorine, mmol/L	103 (101–106)	103 (100–107)	107 (103–111)	< 0.001
TCO2, mmol/L	25 (23–28)	24 (21–28)	21 (15–28)	< 0.001
Haematocrit, %	43 (40–46)	41 (39–44)	41 (40–45)	< 0.001
Haemoglobin, g/dL	14.7 (13.2–15.8)	13.7 (12.8–15.1)	13.9 (12.8–15.7)	< 0.001
Glucose, mg/dL	133 (110–161)	150 (117–219)	196 (140–305)	< 0.001
Lactate, mmol/L	1.96 (1.34–2.47)	2.64 (1.91–4.45)	9.88 (7.82–14.26)	< 0.001
BUN, mg/dL	15 (11–18)	23 (15–35)	36 (24–51)	< 0.001
Creatinine, mg/dL	0.87 (0.75–0.98)	1.15 (0.87–1.54)	1.76 (1.19–2.52)	< 0.001
Urea, mg/dL	32 (24–39)	50 (33–75)	77 (52–110)	< 0.001
Baseline cardiac rhythm				< 0.001
Sinus	349 (66.7)	69 (38.1)	3 (7.5)	
Atrial fibrillation	8 (1.5)	33 (18.2)	8 (20)	
Atrial flutter	0 (0)	0 (0)	1 (2.5)	
Atrial tachycardia	38 (7.3)	22 (22.2)	5 (12.5)	
Supraventricular tachycardia	0 (0)	1 (0.6)	2 (5)	
Ventricular tachycardia	9 (1.7)	8 (4.4)	10 (25)	
Sinus bradycardia	103 (19.7)	30 (16.6)	3 (7.5)	
1°‐degree block	6 (1.1)	4 (2.2)	1 (2.5)	
2° block type I	2 (0.4)	1 (0.6)	0 (0)	
2° block type II	1 (0.2)	1 (0.6)	0 (0)	
Complete block	5 (1)	7 (3.9)	3 (7.5)	
Pacemaker	0 (0)	3 (1.7)	1 (2.5)	
Junctional	2 (0.4)	2 (1.1)	0 (0)	
Idioventricular	0 (0)	0 (0)	3 (7.5)	
Comorbidities				
Heart failure	14 (2.7)	57 (31.5)	6 (15)	< 0.001
Valvular heart disease	15 (2.9)	39 (21.5)	4 (10)	< 0.001
Coagulopathy	8 (1.5)	9 (5)	0 (0)	0.017
Myocardial infarction	122 (23.3)	106 (58.6)	16 (40)	< 0.001
Peripheral vascular disease	63 (12)	53 (29.3)	10 (25)	< 0.001
Hypertension	224 (42.8)	144 (79.6)	25 (62.5)	< 0.001
Cerebrovascular disease	10 (1.9)	24 (13.3)	0 (0)	< 0.001
Chronic pulmonary disease	62 (11.9)	46 (25.4)	10 (25)	< 0.001
Diabetes mellitus	104 (19.9)	87 (48.1)	14 (35)	< 0.001
Hypothyroidism	39 (7.5)	30 (16.6)	7 (17.5)	< 0.001
Chronic renal failure	16 (3.1)	41 (22.7)	7 (17.5)	< 0.001
Liver disease	23 (4.4)	19 (10.5)	4 (10)	0.008
Peptic ulcer disease	36 (6.9)	21 (11.6)	6 (15)	0.045
All cancers	36 (6.9)	33 (18.2)	9 (22.5)	< 0.001
Rheumatoid arthritis	32 (6.1)	38 (21)	3 (7.5)	< 0.001
Obesity	166 (31.7)	68 (37.6)	26 (65)	< 0.001
Alcohol or drug abuse	28 (5.4)	9 (5)	7 (17.5)	0.006
Smoking	182 (34.8)	85 (47)	25 (62.5)	< 0.001
Depression	67 (12.8)	46 (25.4)	8 (20)	< 0.001
Dyslipidemia	284 (54.3)	116 (64.1)	30 (75)	0.005
Connective tissue disease	21 (4)	8 (4.4)	4 (10)	0.209
Dementia	4 (0.8)	23 (12.7)	0 (0)	< 0.001

Abbreviations: BUN, blood urea nitrogen; cSO2, oxygen saturation; NA, not applicable; pCO2, partial pressure of carbon dioxide; pO2, partial pressure of oxygen; SaFi, pulse oximetry saturation/fraction of inspired oxygen ratio; TCO2, total carbon dioxide content; TIMI risk index, Thrombolysis in Myocardial Infarction Risk Index.

^a^ Values expressed as total number (fraction) and medians [25 percentile‐75 percentile], as appropriate.

^b^ The Mann–Whitney *U* test, *T*‐test or chi‐squared test was used as appropriate.

Phenotype P‐2 comprised 24.3%, with 30‐ and 2‐day mortality rates of 22.1% (40 cases) and 12.2% (22 cases), respectively, markedly higher than those of phenotype P‐1. The most frequent location was the anterior vessel with 42% (76 cases), and 33.7% (61 cases) had Killip classification class I (Figure 2A–C). Notably, despite preserved macroscopic hemodynamics (median systolic blood pressure 121 mmHg, heart rate 79 bpm), laboratory parameters revealed evidence of early tissue hypoperfusion: mild acidosis (pH 7.34), hyperlactatemia (2.64 mmol/L), stress hyperglycemia (150 mg/dL), and elevated renal markers (BUN 23 mg/dL, creatinine 1.15 mg/dL). This biochemical profile suggests a state of “cryptic hypoperfusion” or compensated shock—tissue‐level compromise that precedes overt hemodynamic collapse and is not captured by conventional vital sign assessment. Vital signs became unstable, and laboratory tests revealed mild acidosis, hyperlactatemia, and hyperglycemia, with significantly higher advanced life support response rates compared to the P‐1 phenotype, and an OHCA with ROSC rate of 12.2% (22 cases). This cluster is dominated by the prevalence of hypertension, dyslipidemia, history of myocardial infarction, heart failure, diabetes mellitus, and obesity. The median FMC‐to‐balloon time was 68 min (IQR: 47–91).

The P‐3 phenotype comprised 5.4%, with 30‐ and 2‐day mortality rates of 75% (30 cases) and 62.5% (25 cases), respectively. OHCA with ROSC occurred in 87.5% (35 cases), and advanced life support procedures were prominent; for example, prehospital invasive mechanical ventilation was required in 90% (36 cases), and 82.5% (33 cases) required administration of vasoactive agents in the hospital. Vital signs were markedly altered, unequivocally showing signs of hemodynamic instability (desaturation, hypotension, tachycardia, altered level of consciousness), with frank acidosis, severe hyperlactatemia, hyperglycemia, and impaired renal function. The most frequent STEMI location was the anterior vessel in 40% (16 cases), and 67.5% (27 cases) had Killip classification class IV (Figure 2A–C). The most frequent comorbidities were dyslipidemia, hypertension, obesity, tobacco use, history of myocardial infarction, and diabetes mellitus. The median FMC‐to‐balloon time was 79 min (IQR: 65–95). Table 2 shows all analysed outcomes by phenotype. Posthoc comparison for all variables and phenotypes can be found in Table S2.

**TABLE 2:** Prehospital and hospital outcomes of primary coded patients with novel STEMI according to machine‐learning cluster analysis.

	Unsupervised clustering phenotypes			*p* ^b^
	P‐1	P‐2	P‐3	
No. (%) with data^a^	523 (70.3)	181 (24.3)	40 (5.4)	N.A.
Epidemiological data				
STEMI activation criteria				0.056
ST‐segment elevation	480 (91.8)	159 (87.8)	35 (87.5)	
Presumably new branch block	38 (7.3)	15 (8.3)	3 (7.5)	
Ventricular paced rhythm	5 (1)	7 (3.9)	2 (5)	
Rural area	218 (41.7)	89 (49.2)	16 (40)	0.196
Night shift (22:00–07:59)	113 (21.6)	42 (23.2)	8 (20)	0.865
Weekend	135 (25.8)	52 (28.7)	10 (25)	0.729
FMC‐to‐balloon time, min	55 (41–73)	68 (47–91)	79 (65–95)	< 0.001
Prehospital outcomes				
No‐invasive mechanical ventilation	2 (0.4)	18 (9.9)	0 (0)	< 0.001
Invasive mechanical ventilation	10 (1.9)	18 (9.9)	36 (90)	< 0.001
Electrical therapy				< 0.001
Defibrillation	25 (4.8)	17 (9.4)	22 (55)	
Cardioversion	6 (1.1)	2 (1.1)	4 (10)	
External pacemaker	12 (2.3)	7 (3.9)	3 (7.5)	
Drugs administration				
Antiplatelet agent	493 (94.3)	149 (82.3)	16 (40)	< 0.001
Anxiolytic	66 (12.6)	13 (7.2)	0 (0)	0.010
Opioids	297 (56.8)	118 (65.2)	17 (42.5)	0.017
Nitroglycerin	313 (59.8)	75 (41.4)	1 (2.5)	< 0.001
Antiemetic	239 (45.7)	86 (47.5)	1 (2.5)	< 0.001
Antiarrhythmic	30 (5.7)	15 (8.3)	22 (55)	< 0.001
Beta‐blocker	15 (2.9)	10 (5.5)	1 (2.5)	0.231
Vasoactive agents	7 (1.3)	25 (13.8)	22 (55)	< 0.001
Fibrinolysis	6 (1.1)	1 (0.6)	14 (35)	< 0.001
Cardiac arrest	26 (5)	22 (12.2)	35 (87.5)	< 0.001
Hospital outcomes				
Fibrinolysis	13 (2.5)	12 (6.6)	12 (30)	< 0.001
PCI	506 (96.7)	172 (95)	39 (97.5)	0.525
Emergency surgery	3 (0.6)	8 (4.4)	1 (2.5)	0.002
No‐invasive mechanical ventilation	8 (1.5)	22 (12.2)	3 (7.5)	< 0.001
Invasive mechanical ventilation	26 (5)	45 (24.9)	37 (92.5)	< 0.001
Vasoactive agents	42 (8)	66 (36.5)	33 (82.5)	< 0.001
Primary STEMI location				0.874
Anterior	211 (40.3)	76 (42)	16 (40)	
Septal	7 (1.3)	3 (1.7)	3 (7.5)	
Lateral	59 (11.3)	16 (8.8)	2 (5)	
Inferior	219 (41.9)	66 (36.5)	10 (25)	
Posterior	27 (5.2)	20 (11)	9 (22.5)	
Multivessel involvement				< 0.001
Single‐vessel	411 (78.6)	119 (65.7)	13 (32.5)	
Two‐vessel	94 (18)	43 (23.8)	14 (35)	
Three‐vessel	18 (3.4)	19 (10.5)	13 (32.5)	
Killip classification				< 0.001
1 (no failure)	369 (70.6)	61 (33.7)	1 (2.5)	
2 (mild failure)	79 (15.1)	39 (21.5)	6 (15)	
3 (pulmonary edema)	23 (4.4)	31 (17.1)	6 (15)	
4 (cardiogenic shock)	25 (4.8)	43 (23.8)	27 (67.5)	
Length of hospital stay, days	5 (3–7)	7 (4–14)	2 (1–11)	< 0.001
2‐day mortality	7 (1.3)	22 (12.2)	25 (62.5)	< 0.001
30‐day mortality	18 (3.4)	40 (22.1)	30 (75)	< 0.001

Abbreviations: FMC, first medical contact; NA, not applicable; PCI, percutaneous coronary intervention.

^a^ Values expressed as total number (fraction) and medians [25 percentile‐75 percentile], as appropriate.

^b^ The Mann–Whitney *U* test, *T*‐test or chi‐squared test was used as appropriate.

**FIGURE 3:**
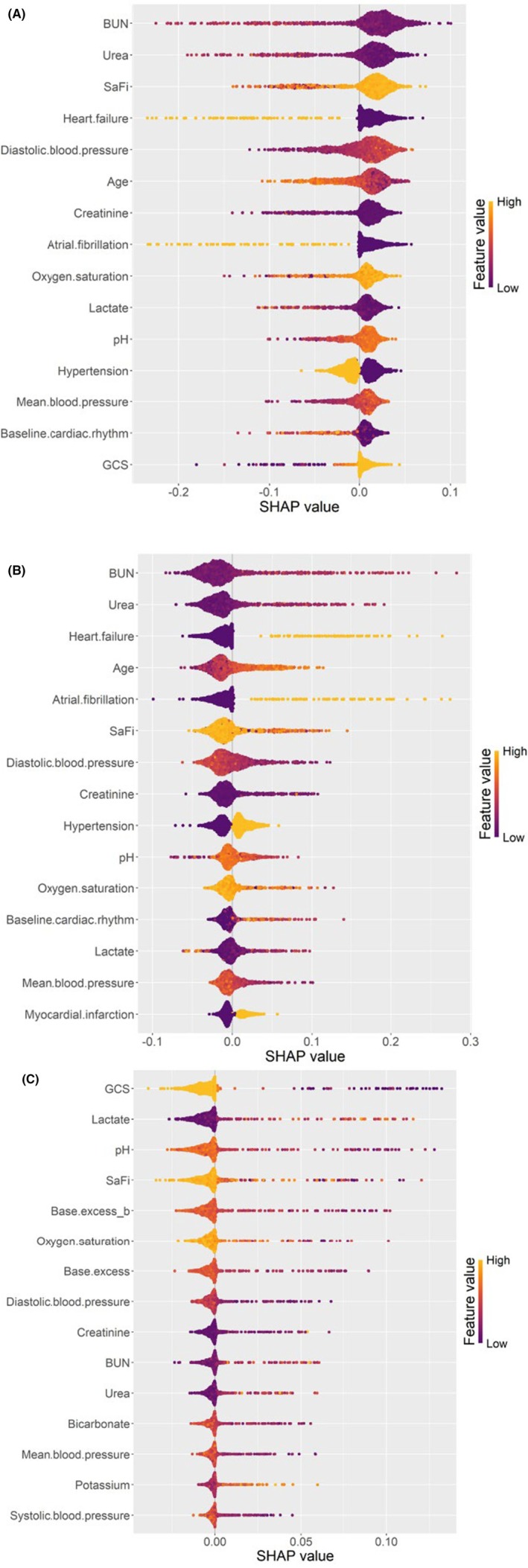
SHapley Additive exPlanations (SHAP) analyses for the Random Forest model trained on the k‐means–derived clusters. (A) P‐1, (B) P‐2, (C) P‐3. In the SHAP summary plot, colour represents the original feature value for each observation, while the x‐axis indicates the contribution of that feature to the predicted probability of cluster membership.

The most important variables for each cluster according to SHAP were BUN, urea, SaFi, and heart failure for P‐1 (Figure 3A), BUN, urea, heart failure, and age for P‐2 (Figure 3B), and GCS, lactate, pH, and SaFi for P‐3 (Figure 3C). The three‐cluster structure remained stable across different FAMD dimension selections; details of this analysis can be found in the Supporting Information (Figure S5).

**FIGURE 4:**
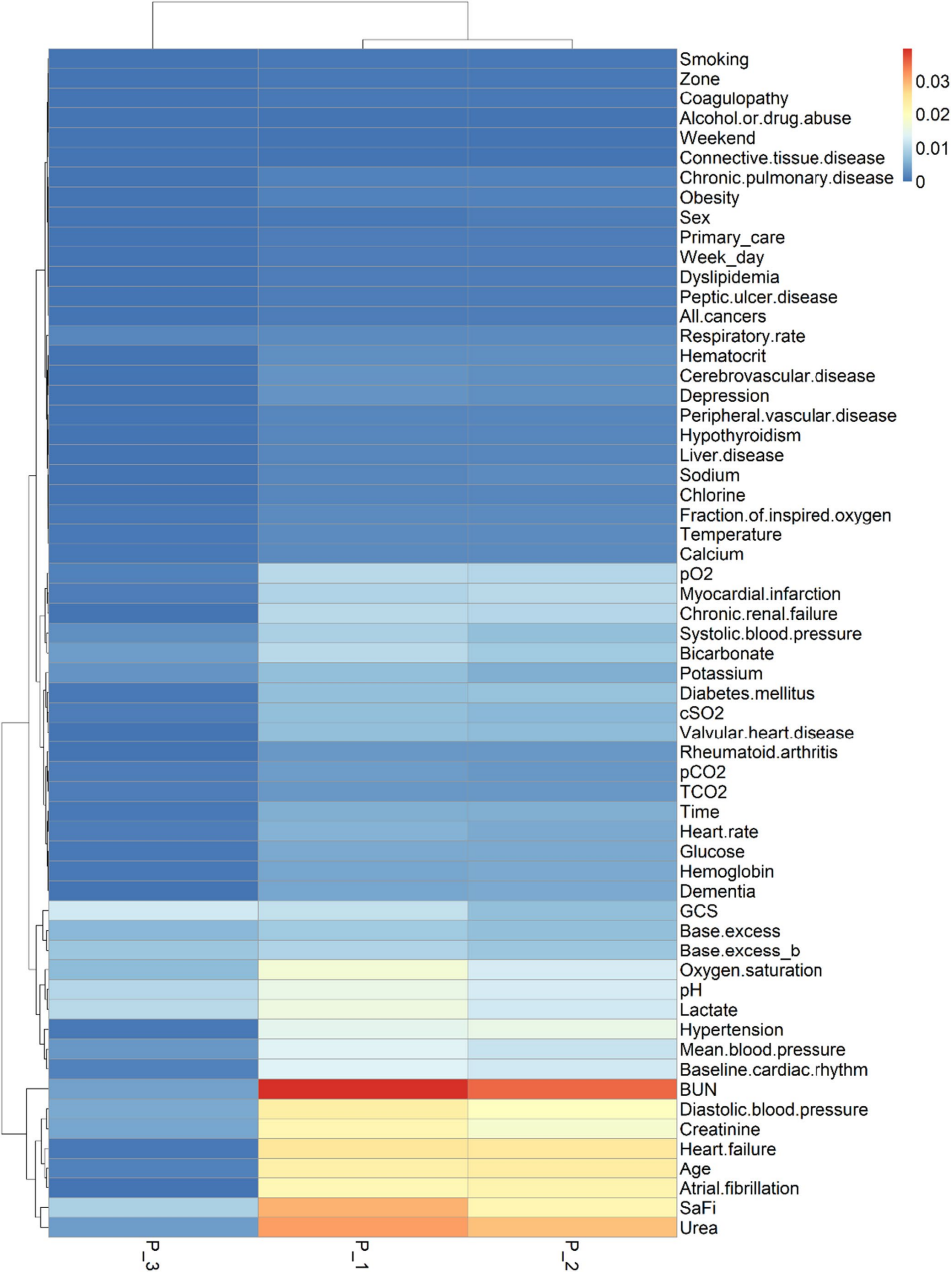
Heatmap of variable importance according to phenotypes and relationship between variables and phenotypes. Higher values (warmer colours) indicate greater influence on predicted cluster membership. Hierarchical clustering was applied to both features and phenotypes to identify shared importance patterns.

This was also shown in Figure 4, representing the variables' importance and the dendrogram with the association between variables. As shown before, variables important for P‐1 and P‐2 were similar, as the phenotypes itself, as shown by the dendrograms.

The model demonstrated high discriminatory performance in reproducing cluster assignments (accuracy > 91.5%, macro F1‐score = 0.81, mean AUC = 0.98 across clusters), indicating that the Random Forest adequately captures the structure of the clustering solution.

Consensus clustering (1000 resampling iterations) was performed to further evaluate robustness. The agreement between the original k‐means solution and the consensus‐derived clusters was excellent, with an Adjusted Rand Index (ARI) of 0.927, indicating near‐perfect concordance, which reflects stability (reproducibility) rather than external validity of the clustering solution.

To explore the OHCA confounder role, the same analyses were performed by excluding OHCA patients (Table S3).

## 4 Discussion

To our knowledge, this is the first study to characterize STEMI phenotypes using unsupervised ML applied exclusively to prehospital clinical, epidemiological, electrocardiographic, and laboratory variables. We identified three distinct phenotypes with progressively worsening outcomes: low‐risk (P‐1, 70.3%), intermediate‐to‐high‐risk (P‐2, 24.3%), and very high‐risk (P‐3, 5.4%). Early prehospital identification of these phenotypes may facilitate targeted risk stratification and guide therapeutic decision‐making to optimize patient outcomes. While clinical characterization of STEMI phenotypes is well established, prehospital perspectives remain underexplored. Previous studies have identified 2–4 distinct clusters using comorbidity patterns [11], lipid profiles [12], hierarchical clustering [13], or myocardial perfusion imaging [14]. Our approach uniquely leverages prehospital variables available at first medical contact, enabling earlier risk stratification than prior phenotyping studies.

Phenotype P‐1, the largest group, comprised the youngest patients with the most favourable outcomes. This low‐risk profile was characterized by predominant Killip class I presentation, inferior vessel localization, single‐vessel involvement, and low incidence of prior cardiovascular events. Traditional modifiable risk factors—dyslipidemia, hypertension, and smoking—were primary drivers [15], consistent with emerging STEMI phenotypes among patients with low baseline cardiovascular risk [13].

Phenotype P‐2 represents the central novel finding of this study and exhibited significantly higher short‐term mortality compared with P‐1, despite presenting with apparent hemodynamic stability at first medical contact. This “occult high‐risk” profile challenges a fundamental assumption in conventional prehospital triage: that initial hemodynamic stability equates to low risk. While mortality gradients between stable and overtly unstable patients are well established, P‐2 reveals a critical blind spot—patients who would not trigger conventional alarm signals yet harbour a six‐fold higher mortality risk than their apparently similar low‐risk counterparts (22.1% vs. 3.4%). Dendrogram analysis revealed structural similarity between these phenotypes, suggesting a continuum of disease severity along a shared pathophysiologic axis. The worse prognosis in P‐2 was driven by increased patient frailty, higher burden of prior cardiovascular disease, greater prevalence of diabetes mellitus with target organ damage [11], and atrial fibrillation [16]. Notably, the P‐2 phenotype represents a clinically crucial subgroup in which absence of heart failure signs at admission does not preclude adverse outcomes. This dissociation between initial Killip class and prognosis may reflect several factors: extent of myocardial damage does not always correlate with initial hemodynamic status [17]; anterior vessel location was the most frequent single location in P‐2 (42%), though the majority of patients presented with non‐anterior locations; nevertheless, anterior involvement when present confers worse prognosis independent of hemodynamic compromise [13]; and compensatory mechanisms may temporarily mask myocardial damage severity. Initial hemodynamic compensation should not provide false reassurance in patients with anterior STEMI and comorbidity burden characteristic of P‐2. The clinical implications of identifying P‐2 are substantial: these patients warrant heightened surveillance, early invasive assessment, and proactive heart failure monitoring despite their reassuring initial presentation. Recognition of this occult high‐risk profile enables targeted interventions before clinical deterioration becomes apparent, potentially reducing the mortality gap between P‐2 and P‐1. Regarding the proportion of patients classified as Killip IV (cardiogenic shock) within P‐1 and P‐2 (4.8% and 23.8% respectively), this finding warrants careful interpretation. Unsupervised clustering assigns patients to phenotypes based on the totality of prehospital variables—not exclusively on hemodynamic status at a single time point. Consequently, a minority of Killip IV patients may be classified into lower‐risk phenotypes if their overall prehospital biochemical and clinical profile more closely resembles that of a given cluster. This reflects the well‐recognized dissociation between the initial Killip classification—which captures a single hemodynamic snapshot—and the multidimensional biological profile captured by the ML approach. In particular, patients presenting with transient or early cardiogenic shock that responds rapidly to prehospital intervention may exhibit a metabolic and clinical profile more consistent with P‐2, emphasizing the importance of a holistic rather than criterion‐based risk assessment. This underscores both a strength and a limitation of phenotype‐based stratification: while it captures complexity beyond isolated parameters, it requires validation against real‐time clinical trajectories. It is noteworthy that after excluding OHCA patients, P‐2 phenotype persisted, and its associated 30‐day mortality rate increased compared with the full cohort analysis.

The pathophysiological basis of the P‐2 phenotype warrants explicit consideration. Despite preserved macroscopic hemodynamics, P‐2 patients exhibited biochemical evidence of early tissue hypoperfusion: elevated lactate, mild acidosis, stress hyperglycemia, and renal marker elevation. This metabolic profile reflects a state of “cryptic hypoperfusion” or compensated shock—a critical transitional phase in which compensatory mechanisms maintain blood pressure while tissue‐level oxygen delivery is already compromised. Standard prehospital vital sign assessment is insensitive to this early compromise; however, point‐of‐care biomarkers can unmask the underlying “biochemical instability” before overt hemodynamic collapse occurs. The SHAP analysis reinforces this interpretation: BUN, urea, heart failure history, and age emerged as the most important discriminating variables for P‐2, reflecting the cardiorenal‐metabolic stress axis that characterizes this occult high‐risk state. These findings underscore the value of prehospital point‐of‐care testing in identifying patients whose risk profile is not captured by conventional clinical assessment.

Phenotype P‐3 represented the highest‐risk group, characterized by cardiogenic shock, OHCA with ROSC and the highest mortality rate, consistent with prior phenotyping studies [18]. This cluster shared key features with the prehospital Early Shock Score high‐risk profile [19], including advanced age, comorbidity burden (heart failure, prior myocardial infarction), anterior STEMI location, hypotension, tachycardia, severe metabolic acidosis, and altered mental status. Both P‐2 and P‐3 were characterized by predominant anterior vessel localization and multivessel involvement. Anterior STEMI location has been associated with increased sympathetic activity, potentially explaining the higher prevalence of arrhythmogenic complications in these higher‐risk phenotypes [20].

This study prospectively evaluated bedside POCT to characterize distinct hemodynamic phenotypes. The P‐3 phenotype demonstrated the most severe metabolic derangements: hyperlactatemia, frank acidosis, and renal failure. Lactate elevation has been associated with significantly increased short‐term mortality in acute cardiovascular disease [21] and emerged as an independent predictor of mortality with predictive capacity comparable to the GRACE 2.0 score in STEMI populations [22]. Prehospital blood glucose levels provided additional prognostic information, demonstrating a stepwise increase across phenotypes correlating directly with mortality, hemodynamic instability, and need for invasive interventions. Admission glucose above 140 mg/dL has been associated with increased risk of death and arrhythmic events in both diabetic and nondiabetic patients [23, 24]. BUN elevation was prominent in P‐2 and P‐3. Given cardiorenal interaction in STEMI, BUN may serve as an early warning marker even when serum creatinine remains normal, identifying patients at heightened risk despite preserved glomerular filtration rate [25].

Phenotype P‐3 had the longest first medical contact‐to‐balloon time, approaching the 90‐min guideline threshold despite remaining within recommended ranges [9, 12]. This delay was attributable to complex prehospital management requirements—cardiogenic shock and OHCA with ROSC necessitated extensive on‐scene stabilization before catheterization laboratory activation. This paradox, where critically ill patients experience longer times to reperfusion, is well‐documented [26, 27] and reflects a gap between guideline recommendations and clinical reality. Atypical presentations and limited initial clinical information further contribute to decision‐making delays. Future strategies should include simultaneous catheterization laboratory activation during stabilization procedures, early mechanical circulatory support team notification for suspected P‐3 patients, and protocol refinements to minimize unavoidable delays without compromising essential stabilization. Most registries inadequately analyse critically ill populations; this study highlights the need for evidence generation in this high‐risk group.

These phenotypes enable actionable prehospital risk stratification with specific management implications. P‐3 patients may benefit from preemptive mechanical circulatory support activation and direct transport to high‐volume centers with advanced heart failure capabilities. P‐2 patients, despite initial hemodynamic stability, warrant heightened surveillance, early invasive assessment, and proactive heart failure monitoring given their occult high‐risk profile. P‐1 patients may be candidates for streamlined care pathways. Implementation of phenotype‐based triage could optimize resource allocation and improve outcomes through earlier, tailored interventions. Specifically, regarding destination triage, identification of P‐2 at first medical contact may justify bypassing smaller district hospitals in favor of tertiary centers with mechanical circulatory support capabilities on standby, given the propensity for rapid hemodynamic deterioration in this phenotype. Regarding prehospital pharmacology, P‐2 patients should prompt early administration of vasoactive support if systolic blood pressure declines, avoidance of nitrates and diuretics that may precipitate hypotension in borderline compensation, and consideration of antiarrhythmic prophylaxis given the high burden of atrial fibrillation. P‐3 patients require immediate hemodynamic support, with prehospital vasopressors and intubation, while antiplatelet loading should be deferred until hemodynamic stabilization given the risk of further cardiovascular compromise. P‐1 patients, in contrast, can follow standard accelerated pathways with routine antiplatelet and analgesic therapy, and targeted transport to the nearest PCI‐capable center without deviation. These actionable differences underscore that the identified phenotypes are not merely descriptive but provide a decision framework adaptable to real‐world EMS operations.

This study has several limitations. First, the study population included only cases of code STEMI activation in which patients called the emergency number and were assessed on‐scene by EMS, thereby excluding STEMI patients who self‐transported to the ED. Although a substantial proportion of STEMI patients arrive without EMS transport [8]. We attempted to ensure the sample was representative by collecting cases continuously (24/7/365) from urban and rural locations via multiple ambulance stations and HEMS across different EMS systems. However, this exclusion may limit the generalizability of our findings to the broader STEMI population. Second, the data extractors were not blinded. To avoid cross‐contamination, EMS providers were not allowed access to hospital follow‐up data, and ED providers were likewise not allowed access to data collected in prehospital care. Only the principal investigator and the data manager were granted full access to the database and derived phenotypes. Thirdly, POCT provides a strength by incorporating biomarkers into the standard workflow that until recently were exclusively hospital‐based [28]. However, despite this, the use of POCT also presents a limitation, as it requires multi‐level management for EMS implementation, resulting in the limited adoption of these new devices. Fourth, the inclusion of OHCA patients with ROSC warrants consideration. While these patients represent an integral component of the real‐world STEMI spectrum that EMS providers must manage, circulatory arrest transforms the clinical scenario from a purely cardiac event to a multi‐organ problem involving post‐cardiac arrest syndrome. Consequently, the P‐3 phenotype findings should be interpreted in this context. Fifth, this study focused exclusively on STEMI, excluding NSTEMI despite the latter encompassing high‐risk presentations such as left main disease. Sixth, sex representation in our cohort reflects the known epidemiology of STEMI, in which women are underrepresented (23.8% in the present study). Sex did not emerge as a significant variable in cluster assignment, and the proportion of women across phenotypes was not statistically different (*p* = 0.051). Nevertheless, given the recognized sex‐specific differences in STEMI presentation, management, and outcomes—including higher rates of atypical symptoms, delayed presentation, and worse prognosis in women—a dedicated sex‐stratified analysis is warranted. Post hoc exploratory analyses stratified by sex did not reveal differential phenotype distributions, though the sample size in P‐3 was insufficient to draw robust conclusions. Future prospective studies should intentionally oversample female STEMI patients to examine whether phenotype‐based stratification performs equitably across sexes. Seventh, data on left ventricular ejection fraction (LVEF) and use of mechanical circulatory support (MCS) during hospitalization were not systematically available for the full cohort. Including LVEF as a hospital outcome would have substantially enhanced the characterization of phenotypic severity; this is acknowledged as a limitation and will be incorporated in future iterations of the APPS study. Finally, external validation is a key component of machine learning models evaluation. As this study focuses on unsupervised phenotyping within a prospective cohort, external validation was not feasible within the current dataset.

In short, unsupervised ML identified three STEMI phenotypes with divergent mortality (3.4%, 22.1%, 75%). Prehospital phenotype‐based stratification facilitates targeted interventions—preemptive mechanical support, specialized center transport, and intensified monitoring—representing a precision medicine approach that optimizes resource allocation and potentially improves time‐sensitive STEMI outcomes.

## Author Contributions

A.R.R. and F.M.‐R. conceptualized the project, managed, and coordinated the project, assisted with the design of the methodology, analysed the data, and prepared the initial and final drafts of the manuscript. A.S.‐G. and C.P.V. take responsibility for the data and their analysis. M.P.‐M., J.F.D.B., and C.T.‐B. assisted with the management and coordination of the project, assisted with the design of the methodology, and helped review the manuscript. R.L.‐I. conceptualized the project and helped review and comment on the initial and final drafts of the manuscript. All the authors performed a critical review and approved the final manuscript for interpretation of the data and important intellectual input. A.S.‐G. and F.M.‐R. have access to and verify the underlying study data.

## Funding

This work was supported by the Institute of Health Carlos III (Spain) and co‐financed by the European Union [grant number DTS23/00010] for FM‐R. By the MCIN/AEI/10.13039/501100011033 and by the European Union [Grant number PID2024‐160665OA‐I00] for AS‐G. The funder of the study played no role in the study design, data collection, data analysis, data interpretation or writing of the report. The authors had full access to all the data in the study and take ultimate responsibility for the decision to submit it for publication.

## Disclosure

Notation of Prior Abstract Publication/Presentation: This article is original work, has not been published before, and is not being considered for publication elsewhere in its final form in either printed or electronic media. It is not based on any previous communication to a society or meeting.

## Ethics Statement

This study was approved by the Health Research Ethics Board of all participating centers (reference: 23‐PI027, EAG‐PRE‐2024‐01, and C‐740). Details of the study design, statistical analysis plan, and raw data are available online.

## Conflicts of Interest

All signed authors meet the requirements of authorship and declare the absence of potential conflicts of interest. A.R.‐R., R.L.‐I., C.P.V., M.P.‐M., C.T.‐B., JFDB, A.S.‐G., and F.M.‐R. report no conflicts of interest. All authors declare no competing interests. On behalf of the other authors, the corresponding author guarantees the accuracy, transparency, and honesty of the data and information contained in the study, that no relevant information has been omitted, and that all discrepancies between authors have been adequately resolved and described.

## Supporting information


**Figure S1:** Analytical Pipeline for Unsupervised Phenotyping of STEMI Patients.
**Figure S2:** Percentage of explained variances by the dimensions resulted from the principal component analysis used for the dimensionality reduction procedure.
**Figure S3:** Optimal number of clusters.
**Figure S4:** Dendrogram from the Hierarchical Clustering.
**Figure S5:** SHapley Additive exPlanations (SHAP) analyses for the Random Forest model trained on the k‐means–derived clusters. A) P‐1, B) P‐2, C) P‐3 for 5 components of the Factor Analysis of Mixed Data (FAMD) procedure, and D) P‐1, E) P‐2, F) P‐3 for 10 components of the FAMD procedure. In the SHAP summary plot, colour represents the original feature value for each observation, while the x‐axis indicates the contribution of that feature to the predicted probability of cluster membership.
**Table S1:** Formulas for calculating the different parameters used in the baseline evaluation.
**Table S2:** Posthoc comparison for all variables and phenotypes.
**Table S3:** Descriptive table of phenotypes without out‐of‐hospital cardiac arrest patients.

## Data Availability

The data that support the findings of this study are available on request from the corresponding author. The data are not publicly available due to privacy or ethical restrictions.
