# Supplementary material for: Prehospital Risk Stratification Using Unsupervised Machine Learning in STEMI
Source: Eur J Clin Invest. 2026 Apr 24;56:e70214. doi: 10.1111/eci.70214 (PMC13108156; doi:10.1111/eci.70214)
Supplement: Supplementary file 1 — Figure S1: Analytical Pipeline for Unsupervised Phenotyping of STEMI Patients. Figure S2: Percentage of explained variances by the dimensions resulted from the principal component analysis used for the dimensionality reduction procedure. Figure S3: Optimal number of clusters. Figure S4: Dendrogram from the Hierarchical Clustering. Figure S5: SHapley Additive exPlanations (SHAP) analyses for the Random Forest model trained on the k‐means–derived clusters. A) P‐1, B) P‐2, C) P‐3 for 5 components of the Factor Analysis of Mixed Data (FAMD) procedure, and D) P‐1, E) P‐2, F) P‐3 for 10 components of the FAMD procedure. In the SHAP summary plot, colour represents the original feature value for each observation, while the x‐axis indicates the contribution of that feature to the predicted probability of cluster membership. Table S1: Formulas for calculating the different parameters used in the baseline evaluation. Table S2: Posthoc comparison for all variables and phenotypes. Table S3: Descriptive table of phenotypes without out‐of‐hospital cardiac arrest patients. [file ECI-56-e70214-s001.docx]

**Supplementary data**

Table of contents

Supplementary methods…………………………………………………………………3

- Supplementary Note 1: Strengthening the Reporting of Observational Studies in Epidemiology (STROBE) Statement.……...……………………………….…3

- Data collection, missing value handling and sample size calculations………...5

Supplementary Results…………………………………………………………………..6

- Supplementary Table S1. Formulas for calculating the different parameters used in the baseline evaluation…………………………………………………..6

- Supplementary Figure S1: Analytical Pipeline for Unsupervised Phenotyping of STEMI Patients……………………………………………………………….7

- Supplementary Figure S2: Percentage of explained variances by the dimensions……………………………………………………………………….8

- Supplementary Figure S3: Optimal number of clusters…………………..……9

- Supplementary Figure S4: Dendrogram from the Hierarchical Clustering..….10

- Supplementary Table S2: Posthoc comparison for all variables and phenotypes……………………………………………………………………...11

- Supplementary Table S3: Descriptive table of phenotypes without out-of-hospital cardiac arrest patients..………………………………………………...17

- Supplementary Figure S4: SHapley Additive exPlanations (SHAP) analyses for the Random Forest model trained on the k-means–derived clusters…………...23

**Supplementary Methods**

STROBE Statement—checklist of items that should be included in reports of observational studies

|  | Item No | Recommendation | Page  No |
| --- | --- | --- | --- |
| **Title and abstract** | 1 | (*a*) Indicate the study’s design with a commonly used term in the title or the abstract | 1 |
|  |  | (*b*) Provide in the abstract an informative and balanced summary of what was done and what was found | 3 |
| Introduction | | | |
| Background/rationale | 2 | Explain the scientific background and rationale for the investigation being reported | 4 |
| Objectives | 3 | State specific objectives, including any prespecified hypotheses | 4 |
| Methods | | | |
| Study design | 4 | Present key elements of study design early in the paper | 5 |
| Setting | 5 | Describe the setting, locations, and relevant dates, including periods of recruitment, exposure, follow-up, and data collection | 5 |
| Participants | 6 | (*a*) *Cohort study*—Give the eligibility criteria, and the sources and methods of selection of participants. Describe methods of follow-up  *Case-control study*—Give the eligibility criteria, and the sources and methods of case ascertainment and control selection. Give the rationale for the choice of cases and controls  *Cross-sectional study*—Give the eligibility criteria, and the sources and methods of selection of participants | 5 |
|  |  | (*b*) *Cohort study*—For matched studies, give matching criteria and number of exposed and unexposed  *Case-control study*—For matched studies, give matching criteria and the number of controls per case | 5 |
| Variables | 7 | Clearly define all outcomes, exposures, predictors, potential confounders, and effect modifiers. Give diagnostic criteria, if applicable | 6 |
| Data sources/ measurement | 8* | For each variable of interest, give sources of data and details of methods of assessment (measurement). Describe comparability of assessment methods if there is more than one group | *6* |
| Bias | 9 | Describe any efforts to address potential sources of bias | 6, 11 |
| Study size | 10 | Explain how the study size was arrived at | 7 |
| Quantitative variables | 11 | Explain how quantitative variables were handled in the analyses. If applicable, describe which groupings were chosen and why | 6 |
| Statistical methods | 12 | (*a*) Describe all statistical methods, including those used to control for confounding | 6 |
|  |  | (*b*) Describe any methods used to examine subgroups and interactions | 6 |
|  |  | (*c*) Explain how missing data were addressed | 6 |
|  |  | (*d*) *Cohort study*—If applicable, explain how loss to follow-up was addressed  *Case-control study*—If applicable, explain how matching of cases and controls was addressed  *Cross-sectional study*—If applicable, describe analytical methods taking account of sampling strategy | 6 |
|  |  | (*e*) Describe any sensitivity analyses | 6 |

Continued on next page

| Results | | | |
| --- | --- | --- | --- |
| Participants | 13* | (a) Report numbers of individuals at each stage of study—eg numbers potentially eligible, examined for eligibility, confirmed eligible, included in the study, completing follow-up, and analysed | 8 |
|  |  | (b) Give reasons for non-participation at each stage | 8 |
|  |  | (c) Consider use of a flow diagram | 8 |
| Descriptive data | 14* | (a) Give characteristics of study participants (eg demographic, clinical, social) and information on exposures and potential confounders | 8 |
|  |  | (b) Indicate number of participants with missing data for each variable of interest | 8 |
|  |  | (c) *Cohort study*—Summarise follow-up time (eg, average and total amount) | 8 |
| Outcome data | 15* | *Cohort study*—Report numbers of outcome events or summary measures over time | *8* |
|  |  | *Case-control study—*Report numbers in each exposure category, or summary measures of exposure | *8* |
|  |  | *Cross-sectional study—*Report numbers of outcome events or summary measures | *8* |
| Main results | 16 | (*a*) Give unadjusted estimates and, if applicable, confounder-adjusted estimates and their precision (eg, 95% confidence interval). Make clear which confounders were adjusted for and why they were included | 8 |
|  |  | (*b*) Report category boundaries when continuous variables were categorized | 8 |
|  |  | (*c*) If relevant, consider translating estimates of relative risk into absolute risk for a meaningful time period | 8 |
| Other analyses | 17 | Report other analyses done—eg analyses of subgroups and interactions, and sensitivity analyses | 8 |
| Discussion | | | |
| Key results | 18 | Summarise key results with reference to study objectives | 9,10 |
| Limitations | 19 | Discuss limitations of the study, taking into account sources of potential bias or imprecision. Discuss both direction and magnitude of any potential bias | 11 |
| Interpretation | 20 | Give a cautious overall interpretation of results considering objectives, limitations, multiplicity of analyses, results from similar studies, and other relevant evidence | 9,10 |
| Generalisability | 21 | Discuss the generalisability (external validity) of the study results | 9,10 |
| Other information | | | |
| Funding | 22 | Give the source of funding and the role of the funders for the present study and, if applicable, for the original study on which the present article is based | 2 |

*Give information separately for cases and controls in case-control studies and, if applicable, for exposed and unexposed groups in cohort and cross-sectional studies.

**Note:** An Explanation and Elaboration article discusses each checklist item and gives methodological background and published examples of transparent reporting. The STROBE checklist is best used in conjunction with this article (freely available on the Web sites of PLoS Medicine at http://www.plosmedicine.org/, Annals of Internal Medicine at http://www.annals.org/, and Epidemiology at http://www.epidem.com/). Information on the STROBE Initiative is available at www.strobe-statement.org.

Data collection, missing value handling and sample size calculations

The data were collected and registered in a database generated with the IBM SPSS Statistics for Apple version 20.0 software. (IBM Corp, Armonk, NY, USA). The caseload entry system was tested to delete unclear or ambiguous values and to verify the adequacy of the data gathering system. Missing values were random; therefore, a listwise deletion method was used since it does not induce biased means, variances or regression weight modifications. The sample size needed for the clustering studies has been recently estimated (1). Due to the characteristics of the clustering procedure, the phenotypes derived from clustering are driven by large effect sizes or by the accumulation of small effect sizes among the multiple variables analyzed, and there is no effect of the covariance structure difference. Therefore, a small sample size (e.g., N=20), as stated in (1), allows large cluster separations.

1-Dalmaijer ES, Nord CL, Astle DE. Statistical power for cluster analysis. BMC Bioinformatics 23, 205 (2022).

Supplementary Table S1. Formulas for calculating the different parameters used in the baseline evaluation

| SaFi | SpO_2_ / FiO_2_ |
| --- | --- |
| MBP | DBP + (SBP - DBP) / 3 |
| TRI | HR × (age / 10)² / SBP |

*Abbreviations*: SaFi: pulse oximetry saturation/fraction of inspired oxygen ratio; SpO_2_: oxygen saturation; FiO_2_: fraction of inspired oxygen; MBP: mean blood pressure; DBP: diastolic blood pressure; SBP: systolic blood pressure; TRI: thrombolysis in myocardial infarction (TIMI) risk index; HR: heart rate.

Supplementary Figure 1. Analytical Pipeline for Unsupervised Phenotyping of STEMI Patients


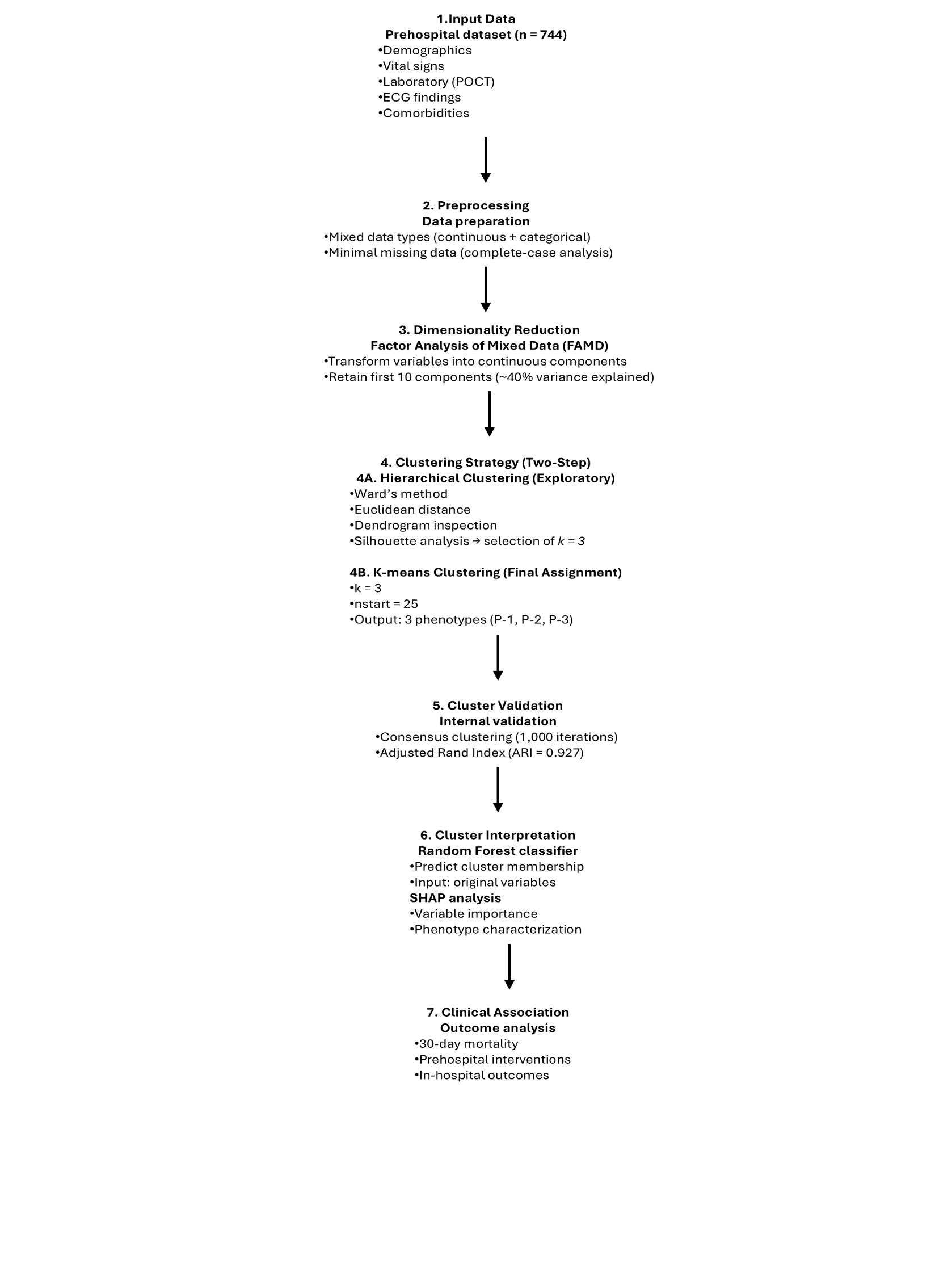


Supplementary Figure 2. Percentage of explained variances by the dimensions resulted from the principal component analysis used for the dimensionality reduction procedure


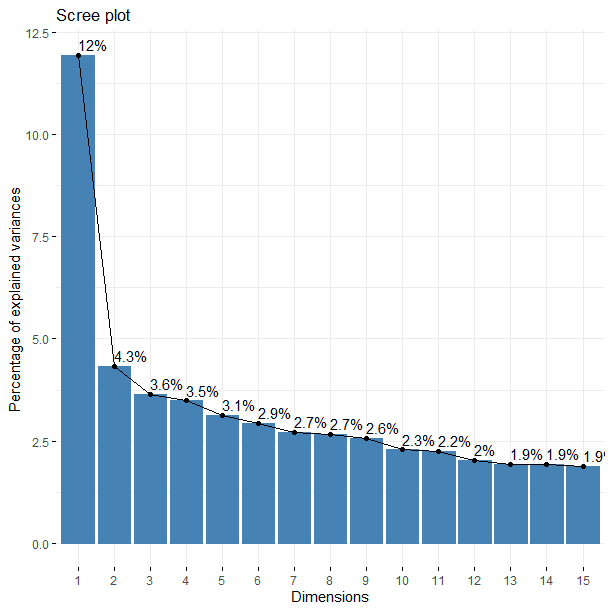


Supplementary Figure 3. Optimal number of clusters

**
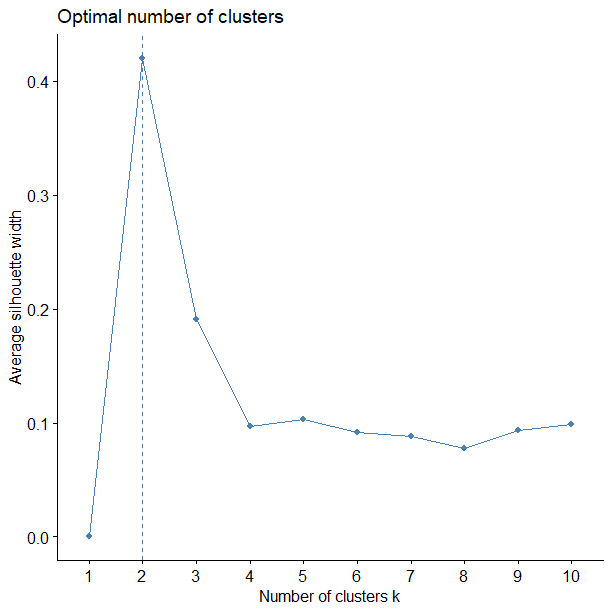
**

Supplementary Figure 4. Dendrogram from the Hierarchical Clustering

**
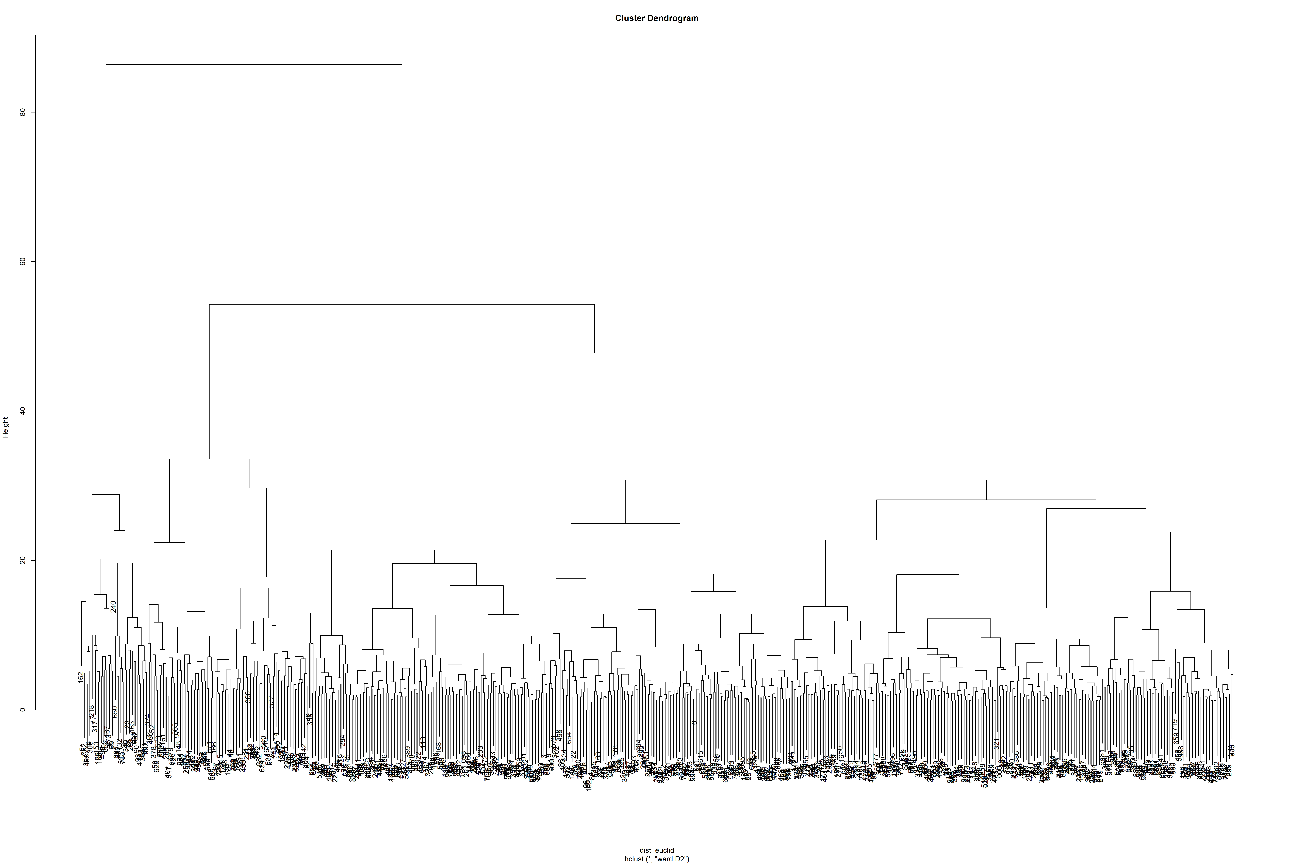
**

Supplementary Table S2: Posthoc comparison for all variables and phenotypes

| **Variable** | **Comparison** | **Adjusted pvalue** |
| --- | --- | --- |
| Age | P_1 - P_2 | <0,001 |
| Age | P_1 - P_3 | 0,024 |
| Age | P_2 - P_3 | 0,005 |
| Respiratory.rate | P_1 - P_2 | 0,550 |
| Respiratory.rate | P_1 - P_3 | <0,001 |
| Respiratory.rate | P_2 - P_3 | <0,001 |
| Oxygen.saturation | P_1 - P_2 | <0,001 |
| Oxygen.saturation | P_1 - P_3 | <0,001 |
| Oxygen.saturation | P_2 - P_3 | <0,001 |
| Fraction.of.inspired.oxygen | P_1 - P_2 | <0,001 |
| Fraction.of.inspired.oxygen | P_1 - P_3 | <0,001 |
| Fraction.of.inspired.oxygen | P_2 - P_3 | <0,001 |
| SaFi | P_1 - P_2 | <0,001 |
| SaFi | P_1 - P_3 | <0,001 |
| SaFi | P_2 - P_3 | <0,001 |
| Systolic.blood.pressure | P_1 - P_2 | <0,001 |
| Systolic.blood.pressure | P_1 - P_3 | <0,001 |
| Systolic.blood.pressure | P_2 - P_3 | <0,001 |
| Diastolic.blood.pressure | P_1 - P_2 | <0,001 |
| Diastolic.blood.pressure | P_1 - P_3 | <0,001 |
| Diastolic.blood.pressure | P_2 - P_3 | <0,001 |
| Mean.blood.pressure | P_1 - P_2 | <0,001 |
| Mean.blood.pressure | P_1 - P_3 | <0,001 |
| Mean.blood.pressure | P_2 - P_3 | <0,001 |
| Heart.rate | P_1 - P_2 | 0,053 |
| Heart.rate | P_1 - P_3 | <0,001 |
| Heart.rate | P_2 - P_3 | 0,011 |
| Temperature | P_1 - P_2 | 0,044 |
| Temperature | P_1 - P_3 | 0,230 |
| Temperature | P_2 - P_3 | 0,892 |
| GCS | P_1 - P_2 | <0,001 |
| GCS | P_1 - P_3 | <0,001 |
| GCS | P_2 - P_3 | <0,001 |
| TIMI risk index | P_1 - P_2 | <0,001 |
| TIMI risk index | P_1 - P_3 | <0,001 |
| TIMI risk index | P_2 - P_3 | <0,001 |
| pH | P_1 - P_2 | <0,001 |
| pH | P_1 - P_3 | <0,001 |
| pH | P_2 - P_3 | <0,001 |
| pCO2 | P_1 - P_2 | <0,001 |
| pCO2 | P_1 - P_3 | <0,001 |
| pCO2 | P_2 - P_3 | <0,001 |
| pO2 | P_1 - P_2 | <0,001 |
| pO2 | P_1 - P_3 | <0,001 |
| pO2 | P_2 - P_3 | <0,001 |
| Bicarbonate | P_1 - P_2 | <0,001 |
| Bicarbonate | P_1 - P_3 | <0,001 |
| Bicarbonate | P_2 - P_3 | <0,001 |
| Base.excess | P_1 - P_2 | <0,001 |
| Base.excess | P_1 - P_3 | <0,001 |
| Base.excess | P_2 - P_3 | <0,001 |
| cSO2 | P_1 - P_2 | <0,001 |
| cSO2 | P_1 - P_3 | <0,001 |
| cSO2 | P_2 - P_3 | <0,001 |
| Potassium | P_1 - P_2 | 0,009 |
| Potassium | P_1 - P_3 | <0,001 |
| Potassium | P_2 - P_3 | <0,001 |
| Chlorine | P_1 - P_2 | 0,768 |
| Chlorine | P_1 - P_3 | <0,001 |
| Chlorine | P_2 - P_3 | 0,001 |
| TCO2 | P_1 - P_2 | 0,286 |
| TCO2 | P_1 - P_3 | 0,001 |
| TCO2 | P_2 - P_3 | 0,004 |
| Hematocrit | P_1 - P_2 | 0,001 |
| Hematocrit | P_1 - P_3 | 0,536 |
| Hematocrit | P_2 - P_3 | 0,356 |
| Hemoglobin | P_1 - P_2 | <0,001 |
| Hemoglobin | P_1 - P_3 | 0,152 |
| Hemoglobin | P_2 - P_3 | 0,491 |
| Glucose | P_1 - P_2 | <0,001 |
| Glucose | P_1 - P_3 | <0,001 |
| Glucose | P_2 - P_3 | 0,009 |
| Lactate | P_1 - P_2 | <0,001 |
| Lactate | P_1 - P_3 | <0,001 |
| Lactate | P_2 - P_3 | <0,001 |
| BUN | P_1 - P_2 | <0,001 |
| BUN | P_1 - P_3 | <0,001 |
| BUN | P_2 - P_3 | 0,002 |
| Urea | P_1 - P_2 | <0,001 |
| Urea | P_1 - P_3 | <0,001 |
| Urea | P_2 - P_3 | 0,002 |
| Creatinine | P_1 - P_2 | <0,001 |
| Creatinine | P_1 - P_3 | <0,001 |
| Creatinine | P_2 - P_3 | <0,001 |
| Baseline.cardiac.rhythm = Sinus | P_1 - P_2 | <0,001 |
| Baseline.cardiac.rhythm = Sinus | P_1 - P_3 | <0,001 |
| Baseline.cardiac.rhythm = Sinus | P_2 - P_3 | 0,001 |
| Baseline.cardiac.rhythm = Atrial tachycardia | P_1 - P_2 | 0,034 |
| Baseline.cardiac.rhythm = Atrial tachycardia | P_1 - P_3 | 0,034 |
| Baseline.cardiac.rhythm = Atrial tachycardia | P_2 - P_3 | 0,425 |
| Baseline.cardiac.rhythm = Sinus bradycardia | P_1 - P_2 | 0,356 |
| Baseline.cardiac.rhythm = Sinus bradycardia | P_1 - P_3 | 0,209 |
| Baseline.cardiac.rhythm = Sinus bradycardia | P_2 - P_3 | 0,235 |
| Baseline.cardiac.rhythm = Atrial fibrillation | P_1 - P_2 | <0,001 |
| Baseline.cardiac.rhythm = Atrial fibrillation | P_1 - P_3 | <0,001 |
| Baseline.cardiac.rhythm = Atrial fibrillation | P_2 - P_3 | 0,534 |
| Baseline.cardiac.rhythm = Ventricular tachycardia | P_1 - P_2 | 0,049 |
| Baseline.cardiac.rhythm = Ventricular tachycardia | P_1 - P_3 | <0,001 |
| Baseline.cardiac.rhythm = Ventricular tachycardia | P_2 - P_3 | <0,001 |
| Baseline.cardiac.rhythm = Complete block | P_1 - P_2 | 0,020 |
| Baseline.cardiac.rhythm = Complete block | P_1 - P_3 | 0,002 |
| Baseline.cardiac.rhythm = Complete block | P_2 - P_3 | 0,119 |
| Heart.failure | P_1 - P_2 | <0,001 |
| Heart.failure | P_1 - P_3 | 0,001 |
| Heart.failure | P_2 - P_3 | 0,042 |
| Valvular.heart.disease | P_1 - P_2 | <0,001 |
| Valvular.heart.disease | P_1 - P_3 | 0,037 |
| Valvular.heart.disease | P_2 - P_3 | 0,104 |
| Coagulopathy | P_1 - P_2 | 0,042 |
| Coagulopathy | P_1 - P_3 | 0,989 |
| Coagulopathy | P_2 - P_3 | 0,989 |
| Myocardial.infarction | P_1 - P_2 | <0,001 |
| Myocardial.infarction | P_1 - P_3 | 0,031 |
| Myocardial.infarction | P_2 - P_3 | 0,035 |
| Peripheral.vascular.disease | P_1 - P_2 | <0,001 |
| Peripheral.vascular.disease | P_1 - P_3 | 0,033 |
| Peripheral.vascular.disease | P_2 - P_3 | 0,588 |
| Hypertension | P_1 - P_2 | <0,001 |
| Hypertension | P_1 - P_3 | 0,024 |
| Hypertension | P_2 - P_3 | 0,024 |
| Cerebrovascular.disease | P_1 - P_2 | <0,001 |
| Cerebrovascular.disease | P_1 - P_3 | 0,989 |
| Cerebrovascular.disease | P_2 - P_3 | 0,989 |
| Chronic.pulmonary.disease | P_1 - P_2 | <0,001 |
| Chronic.pulmonary.disease | P_1 - P_3 | 0,030 |
| Chronic.pulmonary.disease | P_2 - P_3 | 0,957 |
| Diabetes.mellitus | P_1 - P_2 | <0,001 |
| Diabetes.mellitus | P_1 - P_3 | 0,040 |
| Diabetes.mellitus | P_2 - P_3 | 0,136 |
| Hypothyroidism | P_1 - P_2 | 0,002 |
| Hypothyroidism | P_1 - P_3 | 0,046 |
| Hypothyroidism | P_2 - P_3 | 0,887 |
| Chronic.renal.failure | P_1 - P_2 | <0,001 |
| Chronic.renal.failure | P_1 - P_3 | <0,001 |
| Chronic.renal.failure | P_2 - P_3 | 0,476 |
| Liver.disease | P_1 - P_2 | 0,011 |
| Liver.disease | P_1 - P_3 | 0,181 |
| Liver.disease | P_2 - P_3 | 0,926 |
| Peptic.ulcer.disease | P_1 - P_2 | 0,101 |
| Peptic.ulcer.disease | P_1 - P_3 | 0,101 |
| Peptic.ulcer.disease | P_2 - P_3 | 0,554 |
| All.cancers | P_1 - P_2 | <0,001 |
| All.cancers | P_1 - P_3 | 0,002 |
| All.cancers | P_2 - P_3 | 0,534 |
| Rheumatoid.arthritis | P_1 - P_2 | <0,001 |
| Rheumatoid.arthritis | P_1 - P_3 | 0,728 |
| Rheumatoid.arthritis | P_2 - P_3 | 0,088 |
| Obesity | P_1 - P_2 | 0,152 |
| Obesity | P_1 - P_3 | <0,001 |
| Obesity | P_2 - P_3 | 0,003 |
| Alcohol.or.drug.abuse | P_1 - P_2 | 0,843 |
| Alcohol.or.drug.abuse | P_1 - P_3 | 0,012 |
| Alcohol.or.drug.abuse | P_2 - P_3 | 0,014 |
| Smoking | P_1 - P_2 | 0,004 |
| Smoking | P_1 - P_3 | <0,001 |
| Smoking | P_2 - P_3 | 0,004 |
| Depression | P_1 - P_2 | <0,001 |
| Depression | P_1 - P_3 | 0,303 |
| Depression | P_2 - P_3 | 0,472 |
| Dyslipidemia | P_1 - P_2 | 0,034 |
| Dyslipidemia | P_1 - P_3 | 0,034 |
| Dyslipidemia | P_2 - P_3 | 0,190 |
| Dementia | P_1 - P_2 | <0,001 |
| Dementia | P_1 - P_3 | 0,989 |
| Dementia | P_2 - P_3 | 0,989 |
| FMC-to-balloon.time | P_1 - P_2 | <0,001 |
| FMC-to-balloon.time | P_1 - P_3 | <0,001 |
| FMC-to-balloon.time | P_2 - P_3 | 0,034 |
| No-invasive mechanical ventilation | P_1 - P_2 | <0,001 |
| No-invasive mechanical ventilation | P_1 - P_3 | 0,990 |
| No-invasive mechanical ventilation | P_2 - P_3 | 0,990 |
| Invasive mechanical ventilation | P_1 - P_2 | <0,001 |
| Invasive mechanical ventilation | P_1 - P_3 | <0,001 |
| Invasive mechanical ventilation | P_2 - P_3 | <0,001 |
| Defibrillation | P_1 - P_2 | 0,963 |
| Defibrillation | P_1 - P_3 | 0,002 |
| Defibrillation | P_2 - P_3 | 0,014 |
| Cardioversion | P_1 - P_2 | 0,326 |
| Cardioversion | P_1 - P_3 | 0,190 |
| Cardioversion | P_2 - P_3 | 0,326 |
| External pacemaker | P_1 - P_2 | 0,027 |
| External pacemaker | P_1 - P_3 | <0,001 |
| External pacemaker | P_2 - P_3 | <0,001 |
| Antiplatelet agent | P_1 - P_2 | <0,001 |
| Antiplatelet agent | P_1 - P_3 | <0,001 |
| Antiplatelet agent | P_2 - P_3 | <0,001 |
| Anxiolytic | P_1 - P_2 | 0,146 |
| Anxiolytic | P_1 - P_3 | 0,981 |
| Anxiolytic | P_2 - P_3 | 0,981 |
| Opioids | P_1 - P_2 | 0,072 |
| Opioids | P_1 - P_3 | 0,083 |
| Opioids | P_2 - P_3 | 0,027 |
| Nitroglycerin | P_1 - P_2 | <0,001 |
| Nitroglycerin | P_1 - P_3 | <0,001 |
| Nitroglycerin | P_2 - P_3 | 0,001 |
| Antiemetic | P_1 - P_2 | 0,673 |
| Antiemetic | P_1 - P_3 | 0,001 |
| Antiemetic | P_2 - P_3 | 0,001 |
| Antiarrhythmic | P_1 - P_2 | 0,229 |
| Antiarrhythmic | P_1 - P_3 | <0,001 |
| Antiarrhythmic | P_2 - P_3 | <0,001 |
| Vasoactive agents | P_1 - P_2 | 0,001 |
| Vasoactive agents | P_1 - P_3 | <0,001 |
| Vasoactive agents | P_2 - P_3 | <0,001 |
| Fibrinolysis | P_1 - P_2 | 0,496 |
| Fibrinolysis | P_1 - P_3 | <0,001 |
| Fibrinolysis | P_2 - P_3 | <0,001 |
| Cardiac arrest | P_1 - P_2 | 0,001 |
| Cardiac arrest | P_1 - P_3 | <0,001 |
| Cardiac arrest | P_2 - P_3 | <0,001 |
| Fibrinolysis | P_1 - P_2 | 0,001 |
| Fibrinolysis | P_1 - P_3 | <0,001 |
| Fibrinolysis | P_2 - P_3 | <0,001 |
| Emergency surgery | P_1 - P_2 | 0,007 |
| Emergency surgery | P_1 - P_3 | 0,302 |
| Emergency surgery | P_2 - P_3 | 0,583 |
| Single-vessel | P_1 - P_2 | 0,001 |
| Single-vessel | P_1 - P_3 | <0,001 |
| Single-vessel | P_2 - P_3 | <0,001 |
| Two-vessel | P_1 - P_2 | 0,137 |
| Two-vessel | P_1 - P_3 | 0,031 |
| Two-vessel | P_2 - P_3 | 0,144 |
| Three-vessel | P_1 - P_2 | 0,001 |
| Three-vessel | P_1 - P_3 | <0,001 |
| Three-vessel | P_2 - P_3 | 0,001 |
| 1 (no failure) | P_1 - P_2 | <0,001 |
| 1 (no failure) | P_1 - P_3 | <0,001 |
| 1 (no failure) | P_2 - P_3 | 0,003 |
| 2 (mild failure) | P_1 - P_2 | 0,163 |
| 2 (mild failure) | P_1 - P_3 | 0,877 |
| 2 (mild failure) | P_2 - P_3 | 0,455 |
| 3 (pulmonary edema) | P_1 - P_2 | <0,001 |
| 3 (pulmonary edema) | P_1 - P_3 | 0,013 |
| 3 (pulmonary edema) | P_2 - P_3 | 0,671 |
| 4 (cardiogenic shock) | P_1 - P_2 | <0,001 |
| 4 (cardiogenic shock) | P_1 - P_3 | <0,001 |
| 4 (cardiogenic shock) | P_2 - P_3 | <0,001 |
| Length of hospital stay, days | P_1 - P_2 | <0,001 |
| Length of hospital stay, days | P_1 - P_3 | 0,003 |
| Length of hospital stay, days | P_2 - P_3 | <0,001 |
| 2-day mortality | P_1 - P_2 | <0,001 |
| 2-day mortality | P_1 - P_3 | <0,001 |
| 2-day mortality | P_2 - P_3 | <0,001 |
| 30-day mortality | P_1 - P_2 | <0,001 |
| 30-day mortality | P_1 - P_3 | <0,001 |
| 30-day mortality | P_2 - P_3 | <0,001 |

Supplementary Table S3: Descriptive table of phenotypes without out-of-hospital cardiac arrest patients

|  | **P_1** | **P_2** | **P_3** | **pvalue** |
| --- | --- | --- | --- | --- |
|  | ***N=317*** | ***N=83*** | ***N=261*** |  |
| Age | 65.1 (12.6) | 76.7 (12.9) | 65.0 (12.1) | <0.001 |
| Sex: |  |  |  | 0.011 |
| Female | 65 (20.5%) | 30 (36.1%) | 68 (26.1%) |  |
| Weekend: |  |  |  | 0.002 |
| No | 219 (69.1%) | 55 (66.3%) | 211 (80.8%) |  |
| Yes | 98 (30.9%) | 28 (33.7%) | 50 (19.2%) |  |
| Rural: |  |  |  | <0.001 |
| Yes | 168 (53.0%) | 36 (43.4%) | 76 (29.1%) |  |
| Respiratory rate, breaths/min | 17.0 (4.15) | 20.2 (8.90) | 17.6 (5.68) | <0.001 |
| Oxygen saturation, % | 96.6 (2.99) | 90.1 (9.75) | 96.8 (2.55) | <0.001 |
| Fraction of inspired oxygen, % | 0.21 (0.02) | 0.27 (0.16) | 0.21 (0.02) | <0.001 |
| SaFi | 453 (32.9) | 381 (102) | 455 (30.1) | <0.001 |
| Systolic blood pressure, mmHg | 122 (21.4) | 119 (33.2) | 157 (22.5) | <0.001 |
| Diastolic blood pressure, mmHg | 72.6 (13.2) | 67.9 (21.1) | 95.0 (15.6) | <0.001 |
| Mean blood pressure, mmHg | 89.3 (14.5) | 84.7 (24.1) | 116 (15.5) | <0.001 |
| Heart rate, beats/min | 67.5 (17.4) | 86.8 (29.8) | 83.2 (21.7) | <0.001 |
| Temperature, ºC | 36.0 (0.45) | 36.0 (0.76) | 36.0 (0.55) | 0.955 |
| GCS | 14.9 (0.42) | 14.1 (2.64) | 15.0 (0.09) | <0.001 |
| Baseline cardiac rhythm |  |  |  | . |
| Sinus | 182 (57.4%) | 30 (36.1%) | 183 (70.1%) |  |
| Atrial fibrillation | 6 (1.89%) | 16 (19.3%) | 31 (11.9%) |  |
| Atrial flutter | 98 (30.9%) | 9 (10.8%) | 20 (7.66%) |  |
| Atrial tachycardia | 9 (2.84%) | 16 (19.3%) | 15 (5.75%) |  |
| Ventricular tachycardia | 1 (0.32%) | 2 (2.41%) | 7 (2.68%) |  |
| Sinus bradycardia | 17 (5.36%) | 8 (9.64%) | 3 (1.15%) |  |
| 1°-degree block | 2 (0.63%) | 1 (1.20%) | 0 (0.00%) |  |
| Junctional | 2 (0.63%) | 1 (1.20%) | 2 (0.77%) |  |
| pH | 7.38 (0.06) | 7.29 (0.13) | 7.41 (0.07) | <0.001 |
| pCO2, mmHg | 42.4 (9.84) | 49.2 (15.8) | 37.5 (8.61) | <0.001 |
| pO2, mmHg | 29.5 (10.6) | 27.2 (13.7) | 41.9 (19.3) | <0.001 |
| Bicarbonate, mEq | 24.7 (3.60) | 21.1 (4.29) | 23.6 (3.44) | <0.001 |
| Base excess, mmol/L | 0.69 (2.86) | -3.16 (4.81) | -0.23 (3.27) | <0.001 |
| cSO2, % | 46.5 (17.6) | 41.6 (20.3) | 61.4 (20.9) | <0.001 |
| Sodium, mmol/L | 139 (3.28) | 139 (4.54) | 139 (3.69) | 0.693 |
| Potassium, mmol/L | 4.11 (0.59) | 4.46 (1.00) | 4.02 (0.57) | <0.001 |
| Calcium, mmol/L | 1.14 (0.10) | 1.16 (0.12) | 1.14 (0.10) | 0.383 |
| Chlorine, mmol/L | 103 (3.57) | 104 (5.93) | 104 (4.12) | 0.004 |
| TCO2, mmol/L | 26.8 (4.10) | 24.8 (5.15) | 24.8 (4.25) | <0.001 |
| Hematocrit, % | 42.1 (4.63) | 41.6 (6.63) | 43.3 (4.94) | 0.003 |
| Hemoglobin, g/dL | 14.3 (1.84) | 14.0 (2.47) | 14.9 (2.10) | <0.001 |
| Glucose, mg/dL | 137 (45.0) | 218 (102) | 147 (53.1) | <0.001 |
| Lactate, mmol/L | 1.99 (0.84) | 4.51 (3.01) | 2.14 (1.01) | <0.001 |
| BUN, mg/dL | 16.3 (6.60) | 33.9 (15.5) | 16.2 (5.77) | <0.001 |
| Urea, mg/dL | 35.2 (14.1) | 72.7 (33.3) | 34.6 (12.4) | <0.001 |
| Creatinine, mg/dL | 0.91 (0.26) | 1.63 (1.07) | 0.89 (0.26) | <0.001 |
| Heart failure |  |  |  | <0.001 |
| No | 297 (93.7%) | 47 (56.6%) | 247 (94.6%) |  |
| Yes | 20 (6.31%) | 36 (43.4%) | 14 (5.36%) |  |
| Valvular heart disease |  |  |  | <0.001 |
| No | 298 (94.0%) | 60 (72.3%) | 250 (95.8%) |  |
| Yes | 19 (5.99%) | 23 (27.7%) | 11 (4.21%) |  |
| Coagulopathy |  |  |  | 0.351 |
| No | 309 (97.5%) | 79 (95.2%) | 256 (98.1%) |  |
| Yes | 8 (2.52%) | 4 (4.82%) | 5 (1.92%) |  |
| Myocardial infarction |  |  |  | <0.001 |
| No | 230 (72.6%) | 31 (37.3%) | 188 (72.0%) |  |
| Yes | 87 (27.4%) | 52 (62.7%) | 73 (28.0%) |  |
| Peripheral vascular disease |  |  |  | <0.001 |
| No | 270 (85.2%) | 56 (67.5%) | 224 (85.8%) |  |
| Yes | 47 (14.8%) | 27 (32.5%) | 37 (14.2%) |  |
| Hypertension |  |  |  | <0.001 |
| No | 157 (49.5%) | 21 (25.3%) | 138 (52.9%) |  |
| Yes | 160 (50.5%) | 62 (74.7%) | 123 (47.1%) |  |
| Cerebrovascular disease |  |  |  | <0.001 |
| No | 306 (96.5%) | 69 (83.1%) | 256 (98.1%) |  |
| Yes | 11 (3.47%) | 14 (16.9%) | 5 (1.92%) |  |
| Chronic pulmonary disease |  |  |  | <0.001 |
| No | 267 (84.2%) | 57 (68.7%) | 231 (88.5%) |  |
| Yes | 50 (15.8%) | 26 (31.3%) | 30 (11.5%) |  |
| Diabetes mellitus |  |  |  | <0.001 |
| No | 252 (79.5%) | 38 (45.8%) | 193 (73.9%) |  |
| Yes | 65 (20.5%) | 45 (54.2%) | 68 (26.1%) |  |
| Hypothyroidism |  |  |  | 0.123 |
| No | 287 (90.5%) | 69 (83.1%) | 236 (90.4%) |  |
| Yes | 30 (9.46%) | 14 (16.9%) | 25 (9.58%) |  |
| Chronic renal failure |  |  |  | <0.001 |
| No | 294 (92.7%) | 57 (68.7%) | 253 (96.9%) |  |
| Yes | 23 (7.26%) | 26 (31.3%) | 8 (3.07%) |  |
| Liver disease |  |  |  | 0.161 |
| No | 292 (92.1%) | 79 (95.2%) | 250 (95.8%) |  |
| Yes | 25 (7.89%) | 4 (4.82%) | 11 (4.21%) |  |
| Peptic ulcer disease |  |  |  | 0.207 |
| No | 286 (90.2%) | 74 (89.2%) | 245 (93.9%) |  |
| Yes | 31 (9.78%) | 9 (10.8%) | 16 (6.13%) |  |
| All cancers |  |  |  | <0.001 |
| No | 286 (90.2%) | 64 (77.1%) | 242 (92.7%) |  |
| Yes | 31 (9.78%) | 19 (22.9%) | 19 (7.28%) |  |
| Rheumatoid arthritis |  |  |  | 0.002 |
| No | 287 (90.5%) | 66 (79.5%) | 242 (92.7%) |  |
| Yes | 30 (9.46%) | 17 (20.5%) | 19 (7.28%) |  |
| Obesity |  |  |  | 0.136 |
| No | 220 (69.4%) | 48 (57.8%) | 175 (67.0%) |  |
| Yes | 97 (30.6%) | 35 (42.2%) | 86 (33.0%) |  |
| Alcohol or drug abuse |  |  |  | 0.133 |
| No | 294 (92.7%) | 78 (94.0%) | 252 (96.6%) |  |
| Yes | 23 (7.26%) | 5 (6.02%) | 9 (3.45%) |  |
| Smoking |  |  |  | 0.005 |
| No | 207 (65.3%) | 39 (47.0%) | 171 (65.5%) |  |
| Yes | 110 (34.7%) | 44 (53.0%) | 90 (34.5%) |  |
| Depression |  |  |  | 0.009 |
| No | 266 (83.9%) | 60 (72.3%) | 226 (86.6%) |  |
| Yes | 51 (16.1%) | 23 (27.7%) | 35 (13.4%) |  |
| Dyslipidemia |  |  |  | 0.595 |
| No | 143 (45.1%) | 37 (44.6%) | 107 (41.0%) |  |
| Yes | 174 (54.9%) | 46 (55.4%) | 154 (59.0%) |  |
| Connective tissue disease |  |  |  | 0.372 |
| No | 307 (96.8%) | 78 (94.0%) | 249 (95.4%) |  |
| Yes | 10 (3.15%) | 5 (6.02%) | 12 (4.60%) |  |
| Dementia |  |  |  | <0.001 |
| No | 313 (98.7%) | 69 (83.1%) | 254 (97.3%) |  |
| Yes | 4 (1.26%) | 14 (16.9%) | 7 (2.68%) |  |
| TIMI risk index | 24.5 (11.6) | 47.0 (28.3) | 23.9 (12.3) | <0.001 |
| No-invasive mechanical ventilation |  |  |  | <0.001 |
| 0 | 316 (99.7%) | 68 (81.9%) | 258 (98.9%) |  |
| 1 | 1 (0.32%) | 15 (18.1%) | 3 (1.15%) |  |
| Invasive mechanical ventilation |  |  |  | <0.001 |
| 0 | 313 (98.7%) | 75 (90.4%) | 261 (100%) |  |
| 1 | 4 (1.26%) | 8 (9.64%) | 0 (0.00%) |  |
| Electrical therapy |  |  |  | 0.076 |
| Defibrillation | 302 (95.3%) | 75 (90.4%) | 249 (95.4%) |  |
| Cardioversion | 2 (0.63%) | 2 (2.41%) | 4 (1.53%) |  |
| Cardioversion other | 2 (0.63%) | 2 (2.41%) | 5 (1.92%) |  |
| External pacemaker | 11 (3.47%) | 4 (4.82%) | 3 (1.15%) |  |
| Vasoactive |  |  |  | <0.001 |
| 0 | 311 (98.1%) | 67 (80.7%) | 260 (99.6%) |  |
| 1 | 6 (1.89%) | 16 (19.3%) | 1 (0.38%) |  |
| Emergency surgery |  |  |  | 0.008 |
| 0 | 314 (99.1%) | 78 (94.0%) | 259 (99.2%) |  |
| 1 | 3 (0.95%) | 5 (6.02%) | 2 (0.77%) |  |
| PCI |  |  |  | 0.030 |
| 0 | 7 (2.21%) | 7 (8.43%) | 11 (4.21%) |  |
| 1 | 310 (97.8%) | 76 (91.6%) | 250 (95.8%) |  |
| Fibrinolysis |  |  |  | <0.001 |
| 0 | 313 (98.7%) | 74 (89.2%) | 253 (96.9%) |  |
| 1 | 4 (1.26%) | 9 (10.8%) | 8 (3.07%) |  |
| Killip classification |  |  |  | <0.001 |
| 1 (no failure) | 219 (74.2%) | 13 (16.0%) | 190 (75.7%) |  |
| 2 (mild failure) | 50 (16.9%) | 14 (17.3%) | 39 (15.5%) |  |
| 3 (pulmonary edema) | 14 (4.75%) | 22 (27.2%) | 12 (4.78%) |  |
| 4 (cardiogenic shock) | 12 (4.07%) | 32 (39.5%) | 10 (3.98%) |  |
| Primary STEMI location |  |  |  | . |
| Anterior | 106 (33.4%) | 32 (38.6%) | 123 (47.1%) |  |
| Septal | 5 (1.58%) | 1 (1.20%) | 4 (1.53%) |  |
| Lateral | 33 (10.4%) | 11 (13.3%) | 29 (11.1%) |  |
| Inferior | 152 (47.9%) | 28 (33.7%) | 93 (35.6%) |  |
| Posterior | 21 (6.62%) | 11 (13.3%) | 12 (4.60%) |  |
| Multivessel involvement |  |  |  | . |
| Single-vessel | 248 (78.2%) | 52 (62.7%) | 205 (78.5%) |  |
| Two-vessel | 55 (17.4%) | 20 (24.1%) | 50 (19.2%) |  |
| Three-vessel | 14 (4.42%) | 11 (13.3%) | 6 (2.30%) |  |
| Length of hospital stay, days | 6.89 (6.20) | 10.9 (11.6) | 6.51 (10.3) | <0.001 |
| 30-day mortality |  |  |  | <0.001 |
| No | 310 (97.8%) | 48 (57.8%) | 252 (96.6%) |  |
| Yes | 7 (2.21%) | 35 (42.2%) | 9 (3.45%) |  |
| 2-day mortality |  |  |  | <0.001 |
| No | 316 (99.7%) | 62 (74.7%) | 258 (98.9%) |  |
| Yes | 1 (0.32%) | 21 (25.3%) | 3 (1.15%) |  |
|  |  |  |  |  |

Supplementary Figure 5. SHapley Additive exPlanations (SHAP) analyses for the Random Forest model trained on the k-means–derived clusters. A) P-1, B) P-2, C) P-3 for 5 components of the Factor Analysis of Mixed Data (FAMD) procedure, and D) P-1, E) P-2, F) P-3 for 10 components of the FAMD procedure. In the SHAP summary plot, color represents the original feature value for each observation, while the x-axis indicates the contribution of that feature to the predicted probability of cluster membership.

| A) | 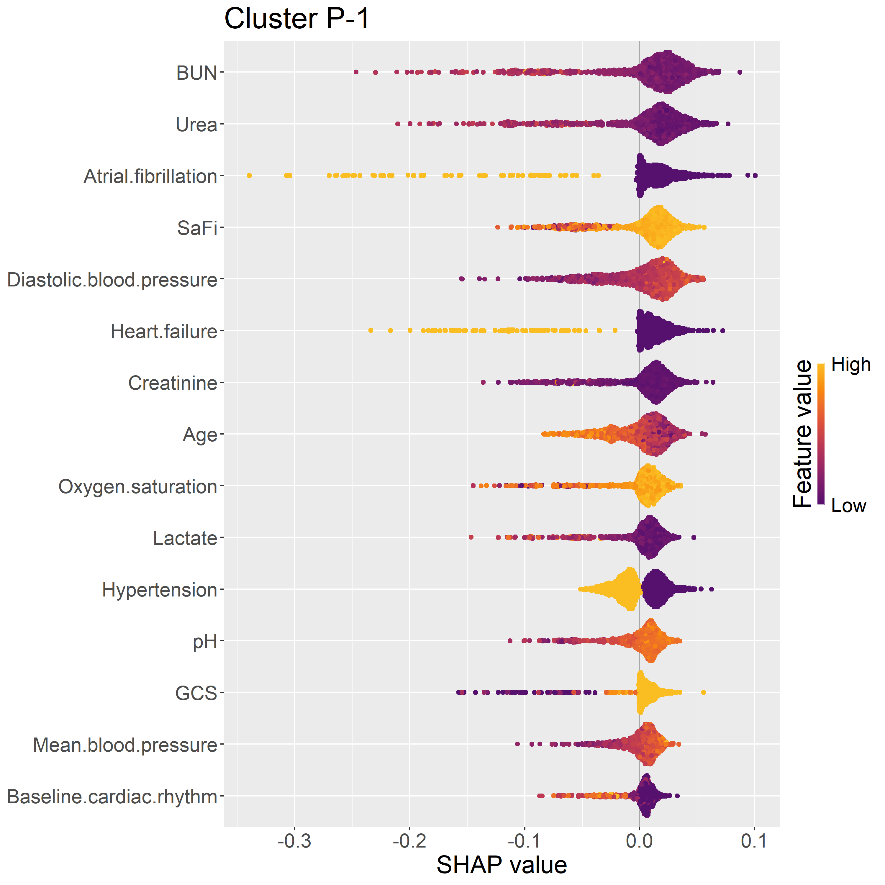 |
| --- | --- |
| B) | 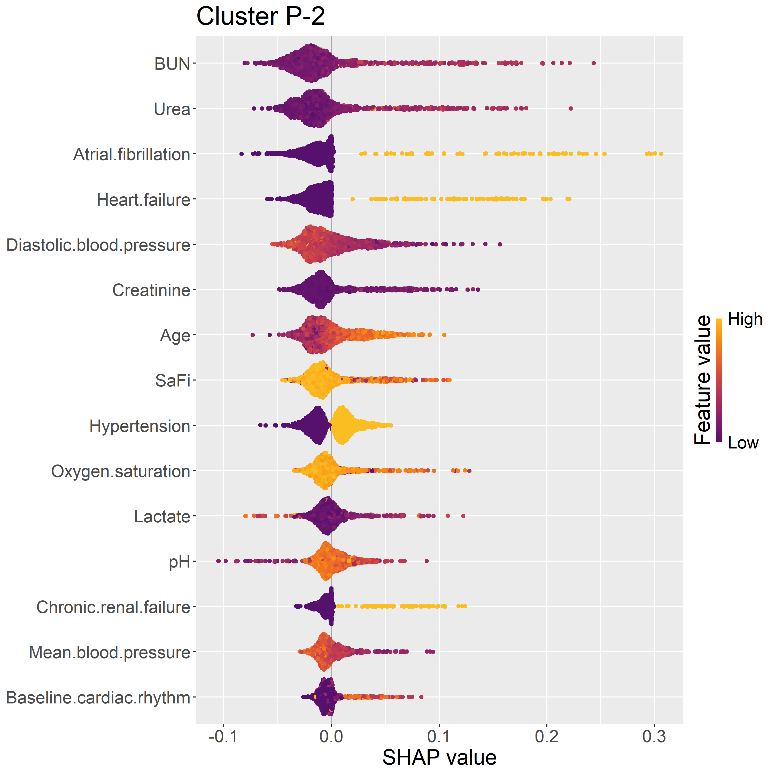 |
| C) | 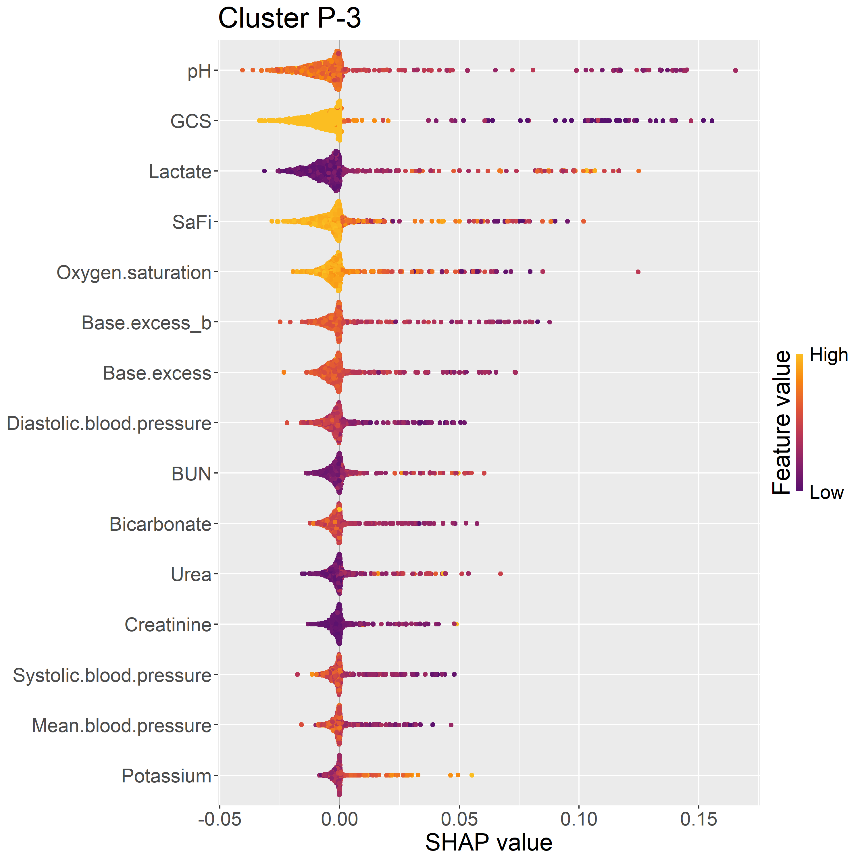 |

D)


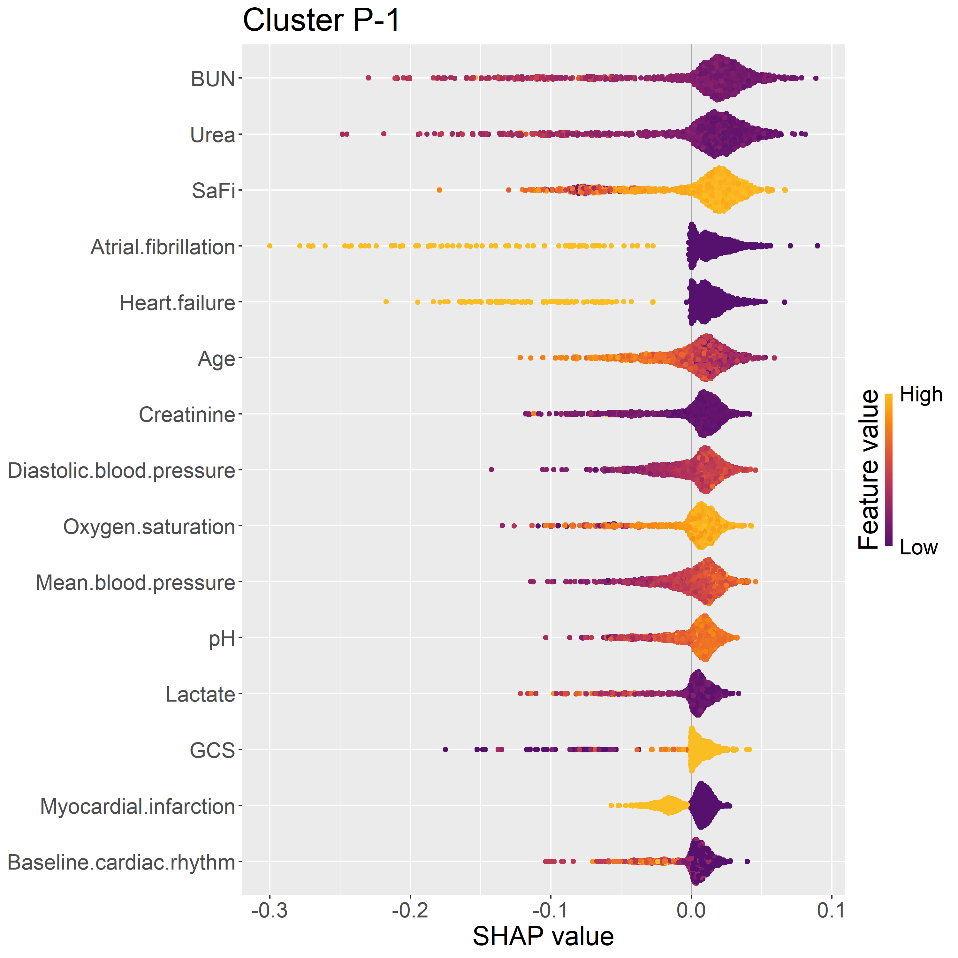


E)


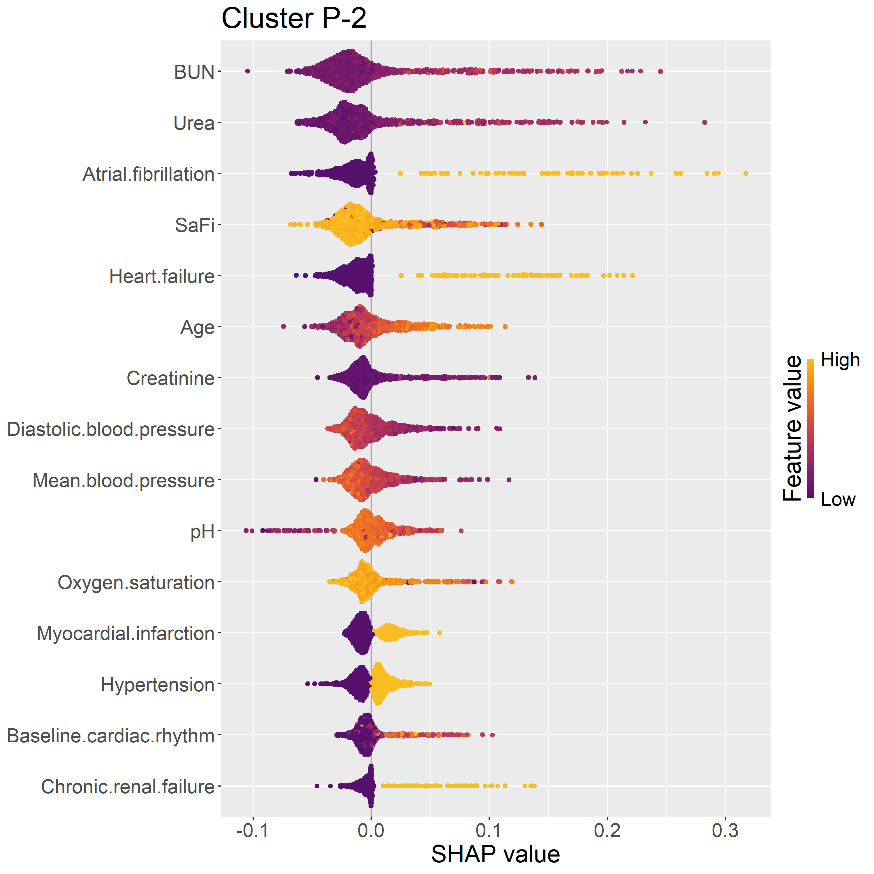


F)

**
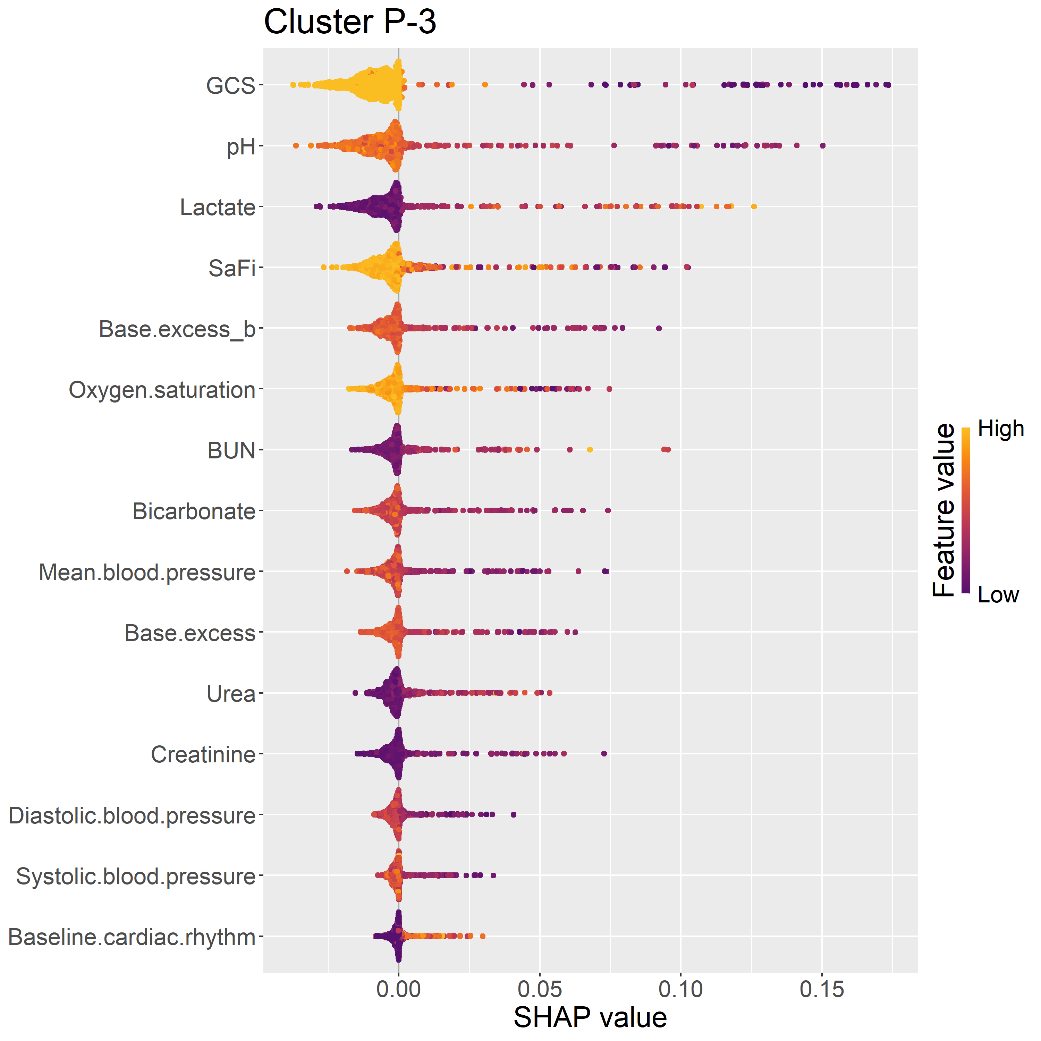
**
